# Determinants of the de-implementation of low-value care: a multi-method study

**DOI:** 10.1186/s12913-022-07827-4

**Published:** 2022-04-06

**Authors:** Jeanna Parsons Leigh, Emma E. Sypes, Sharon E. Straus, Danielle Demiantschuk, Henry Ma, Rebecca Brundin-Mather, Chloe de Grood, Emily A. FitzGerald, Sara Mizen, Henry T. Stelfox, Daniel J. Niven

**Affiliations:** 1 School of Health Administration, Faculty of Health, Dalhousie University, Halifax, Canada; 2 Faculty of Medicine, University of Ottawa, Ottawa, Canada; 3 Li Ka Shing Knowledge Institute of St. Michael’s Hospital, University of Toronto, Toronto, Canada; 4 Research Services, University of Calgary, Calgary, Canada; 5 Faculty of Medicine and Dentistry, University of Alberta, Edmonton, Canada; 6 Department of Critical Care Medicine, Cumming School of Medicine, University of Calgary, Calgary, Canada; 7 Department of Community Health Sciences, Cumming School of Medicine, University of Calgary, Calgary, Canada; 8 O’Brien Institute for Public Health, Cumming School of Medicine, University of Calgary, Calgary, Canada

**Keywords:** de-implementation, de-adoption, low-value care, behaviour change, barriers and facilitators

## Abstract

**Background:**

There is an urgent need to understand the determinants (i.e., barriers and facilitators) of de-implementation. The purpose of this study was to develop a comprehensive list of determinants of the de-implementation of low-value care from the published literature and to compare this list to determinants identified by a group of stakeholders with lived experience with de-implementation.

**Methods:**

This was a two-phase multi-method study. First, a systematic review examined published barriers and facilitators to de-implementation. Articles were identified through searches within electronic databases, reference lists and the grey literature. Citations were screened independently and in duplicate and included if they were: 1) written in English; and 2) described a barrier or facilitator to de-implementation of any clinical practice in adults (age ≥ 18 years). ‘Raw text’ determinants cited within included articles were extracted and synthesized into a list of representative determinants using conventional content analysis. Second, semi-structured interviews were conducted with decision-makers (unit managers and medical directors) and healthcare professionals working in adult critical care medicine to explore the overlap between the determinants found in the systematic review to those experienced in critical care medicine. Thematic content analysis was used to identify key themes emerging from the interviews.

**Results:**

In the systematic review, reviewers included 172 articles from 35,368 unique citations. From 437 raw text barriers and 280 raw text facilitators, content analysis produced 29 distinct barriers and 24 distinct facilitators to de-implementation. Distinct barriers commonly cited within raw text included ‘lack of credible evidence to support de-implementation’ (*n* = 90, 21%), ‘entrenched norms and clinicians’ resistance to change (*n* = 43, 21%), and ‘patient demands and preferences’ (*n* = 28, 6%). Distinct facilitators commonly cited within raw text included ‘stakeholder collaboration and communication’ (*n* = 43, 15%), and ‘availability of credible evidence’ (*n* = 33, 12%). From stakeholder interviews, 23 of 29 distinct barriers and 20 of 24 distinct facilitators from the systematic review were cited as key themes relevant to de-implementation in critical care.

**Conclusions:**

The availability and quality of evidence that identifies a clinical practice as low-value, as well as healthcare professional willingness to change, and stakeholder collaboration are common and important determinants of de-implementation and may serve as targets for future de-implementation initiatives.

**Trial registration:**

The systematic review was registered in PROSPERO CRD42016050234.

**Supplementary Information:**

The online version contains supplementary material available at 10.1186/s12913-022-07827-4.

## Background

Clinical practices that are unnecessary or potentially harmful (i.e., low-value care [1]) expose patients to avoidable risks of harm and are incongruent with global efforts to improve patient-centered care [2]. Low-value care is a source of needless consumption of valuable healthcare resources and contributes to financial instability within healthcare systems [3–5]. Recent estimates suggest that low-value care accounts for millions if not billions of dollars of wasteful healthcare spending within high income countries [6–9]. Not only is low-value care a source of wasteful spending, but it may also be a source of unnecessary medical waste, a phenomenon recently recognized as contributing to global climate change [10].

The recognition that certain aspects of medical care may be low-value has been acknowledged since the early twenty-first century when Fisher et al. suggested that up to 30% of all medical care in the United States may be unnecessary [11, 12]. Other similar estimates subsequently catalyzed numerous initiatives seeking to decrease the use of low-value care (e.g. Choosing Wisely [13]), many of which have arisen within the last 10 years [14]. However, such extensive efforts have not translated into similar decreases in the use of low-value care [15–20]. While this observation has many explanations, one important factor that remains is the lack of understanding of the process of de-implementation and the determinants that enable (i.e., facilitators) and impede (i.e., barriers) its success.

Most research examining determinants of practice change has focused on the implementation of high-value practices [21, 22]. Although similarities likely exist between implementation of high-value care and de-implementation of low-value care, studies suggest that de-implementation is more difficult and may require different, nuanced considerations [23]. Two studies recently examined determinants of reducing low-value care [24, 25]. In a qualitative evidence synthesis that identified 81 articles focused mostly on low-value therapeutics, van Dulmen et al. identified provider, organizational, and patient-related factors as the most common determinants [25]. For providers, most determinants related to their attitude towards de-implementation [25]. For organizations it was having appropriate resources, and for patients it was knowledge of which potentially common practices may be low-value [25]. Using scoping review methodology, Augustsson et al. found that within 101 relevant citations patients’ expectations and professionals’ fear of malpractice were prominent determinants of use and de-implementation of low-value care [24]. Though these two recent evidence syntheses make important contributions to understanding the process of de-implementation, limitations applied to the searches may have missed potentially important determinants. Also, it is unclear how such determinants compare to those identified by stakeholders with lived experience with de-implementation, and whether they represent useful, actionable items that will improve de-implementation efforts. To address this gap, we conducted a two-phase multi-method study to identify determinants of de-implementation within the literature and compare these determinants to those identified by stakeholders within a test medical discipline, namely critical care medicine. This study is part of a broader program of research to develop a framework to guide de-implementation within acute care [26].

## Methods

### Systematic Review

The systematic review builds on a prior scoping review that explored de-implementation of clinical practices in adults with medical, surgical, or psychiatric illnesses [15]. For the current systematic review, we updated the search from the scoping review and restricted our focus to articles that described barriers and facilitators to de-implementation. Determinants of de-implementation were defined as factors that impeded (i.e. barriers) or enabled (i.e. facilitators) the discontinuation of a previously implemented clinical practice (e.g., clinical champions, scientific evidence supporting de-implementation) [15, 27]. We used a model of de-implementation proposed in our scoping review to conceptualize de-implementation and map barriers and facilitators [15]. A protocol for this systematic review was developed a priori and registered with PROSPERO (CRD42016050234) [26], and our methodology was guided by the Joanna Briggs Institute Reviewer’s Manual [28]. We reported our methods and findings in accordance with the Preferred Reporting Items for Systematic Reviews and Meta-Analyses (PRISMA) checklist.

#### Data Sources and Searches

Potentially eligible articles were identified through searches conducted within MEDLINE, EMBASE, CINAHL, the Cochrane Central Register of Controlled Trials (CENTRAL), Cochrane Database of Systematic Reviews, and the Cochrane Database of Abstracts and Reviews of Effects from January 1, 1990 to October 17, 2016. An experienced medical librarian assisted with the development of the search strategy, which was then peer reviewed by a second medical librarian using the Peer Review of Electronic Search Strategies (PRESS) checklist [29]. Our search strategy included key words and synonyms related to de-implementation and clinical practices. The search strategy was developed in MEDLINE (Additional File 1) and modified for other electronic databases. In addition to electronic databases, we searched reference lists of included articles and the grey literature (e.g., http://choosingwisely.org) using the CADTH Grey Literature Search Tool [30].

#### Study Selection

Articles were eligible for inclusion if they: 1) were written in English; and 2) described a determinant of de-implementation of any clinical practice in adults (age ≥ 18 years) with medical, surgical, or psychiatric illnesses. We included articles reporting original (e.g., qualitative and quantitative studies) and non-original (e.g., narrative reviews, editorials) research. The screening form was pilot tested using a random sample of 50 articles and revised until agreement was reliable (kappa ≥0.8). Full article screening was conducted in two stages with two independent reviewers. In Stage 1, reviewers used the screening form to screen titles and abstracts of potentially relevant articles. In Stage 2, the full-text of any article categorized as “include” or “unclear” in Stage 1 was screened to determine final eligibility. Agreement was quantified using the kappa statistic with kappa > 0.8 denoting high-level agreement [31]. Disagreements were resolved by consensus or a third reviewer. Articles were stored and managed using Endnote X7 (Clarivate Analytics, Philadelphia, USA).

#### Data Extraction and Quality Assessment

Two investigators independently extracted data from all included articles using an electronic form that was pilot tested using a random sample of 10 articles until agreement was reliable (kappa ≥0.8). Extracted data pertained to the article’s characteristics (e.g., study design), focus (e.g., identify low-value practices), the targeted low-value practice(s) (e.g., therapeutic interventions), and our primary outcomes of interest, the reported determinants of de-implementation. The in-article text used to describe each reported barrier and/or facilitator served as our raw data for content analysis.

Two investigators evaluated original research articles using the Quality Assessment Tool for Studies with Diverse Designs (QATSDD) [32]. For each article, we calculated a total score and percentage of total score to facilitate comparison across studies with different designs. Disagreements were resolved by a third investigator.

#### Data Synthesis and Analysis

We used conventional content analysis to inductively code raw text from included articles describing barriers and facilitators to develop a representative, list of distinct determinants [33]. For the purposes of this study, distinct determinants were barriers and facilitators that described different concepts relevant to de-implementation. Two investigators began by independently familiarizing themselves with the data and applying initial codes that captured key concepts. To ensure consistency, they iteratively reviewed, compared, and modified codes until a final coding scheme was established [34]. The coding scheme was then applied to the raw text to synthesize the barriers and facilitators into representative groupings. Each raw text barrier and facilitator was counted once per article, and total counts were calculated throughout the coding process. To reflect their frequency of citation, the final list of distinct barriers and facilitators were rank-ordered by number of citations. Two investigators subsequently independently mapped the finalized list to two conceptual frameworks for behavior change: 1) the Theoretical Domains Framework [35]; and 2) a conceptual model for facilitating de-implementation [15]. Two investigators also independently mapped each barrier and facilitator to its most relevant stakeholder category – clinicians, patients, researchers, decision-makers. For the purposes of this study, clinicians included healthcare professionals providing care to patients. Decision-makers included members of the healthcare team responsible for managing and maintaining the healthcare system (e.g. unit managers, medical directors). Each determinant could be mapped to more than one stakeholder category. All data were stored and analyzed in Microsoft Excel (Microsoft, Washington, USA).

### Stakeholder Interviews

#### Overview

A qualitative description study design was used to examine participants’ experiences and insights regarding de-implementation in critical care medicine [36]. There is limited data about de-implementation in critical care, therefore, semi-structured interviews offered an opportunity to elicit perceptions and experiences through open-ended questions and probing. Ethical approval was obtained from The University of Calgary Conjoint Health Research Ethics Board (REB17–2153) and participants provided informed consent prior to being interviewed.

#### Participants

A sample of critical care stakeholders with lived experience with de-implementation including decision-makers (i.e., unit managers, medical directors) and front-line healthcare professionals (i.e., physicians, nurses) from one province (Alberta, Canada) were recruited through purposive (i.e., circles of contact) and snowball sampling. Potential participants were approached through email correspondence and were made aware of the research goals and intentions. Circles of contact included department heads at the University of Calgary and University of Alberta. Contacts sent the recruitment email to their contact lists which included people at four adult Intensive Care Units (ICUs) in Calgary and five adult ICUs in Edmonton. Interview participants also provided contact information to other unit directors, managers, physicians and nurses to facilitate snowball sampling. We aimed to recruit five to seven decision-makers, five to seven physicians and five to seven nurses; however, we continued sampling until data saturation was achieved [37].

#### Data Collection

A semi-structured interview guide was developed iteratively and pilot tested with two critical care stakeholders (Additional File 2). Questions were informed by the Theoretical Domains Framework and focused on the barriers and facilitators to de-implementation. Refinements were made to the interview guide after each pilot test. Semi-structured interviews were conducted from June 24, 2019-Feb 20, 2020. Researchers with advanced training in qualitative research conducted interviews in a private office at the University of Calgary. The interviews were audio recorded, transcribed, de-identified and assigned a unique identifier. Data collection continued until it was determined that thematic saturation was achieved, in which no new themes were identified from participant interviews. Respondents were given the opportunity to review their transcripts for additional comment or correction.

#### Data Analysis

Thematic content analysis was conducted on all interviews in duplicate using NVivo software (Version 12) [38]. Two investigators (EF, SM) began by familiarizing themselves with the data and developed a codebook from the determinants identified in the systematic review. A research meeting was held after the first three transcripts were coded using the developed codebook and questions (e.g., interpreting determinants from the systematic review in the context of critical care) were addressed before moving forward. A follow-up meeting occurred after another two transcripts were coded to ensure similar approach and consistency in coding of the text data (kappa > 0.8) before moving forward with coding remaining transcripts. Consistency in coding was examined after every five transcripts and required an overall kappa > 0.8. We deductively developed our coding and determinant themes to explore common determinants to de-implementation between published literature and critical care medicine. Disagreements were resolved through discussion and a list of determinants were finalized.

## Results

### Systematic Review

The electronic database and grey literature search returned 35,368 unique citations. Of these, 337 warranted full-text review and 172 studies were included in the final systematic review. The most common reason for exclusion after full-text review was no discussion of the determinants of de-implementation (Additional File 3).

#### Characteristics of Included Studies

As outlined in Table 1, articles reporting original research (*n* = 76, 44%) were most commonly cohort (*n* = 25, 15%), quasi-experimental (*n* = 18, 10%), and mixed-methods studies (*n* = 7, 4%). Non-original research (*n* = 96, 56%) included editorials, websites/news items (*n* = 64, 37%), or literature reviews (*n* = 29, 17%). Barriers and facilitators to de-implementation were reported in 80% (*n* = 138) and 61% (*n* = 105) of included studies, respectively. Among articles that described determinants as related to a specific low-value practice, therapeutic interventions (e.g., antibiotics for upper respiratory tract infections) were more common (*n* = 42, 24%) than low-value diagnostic interventions (e.g., imaging for low back pain) (*n* = 33, 19%). Individual study characteristics are presented in Additional File 4.

**Table 1:** Characteristics of studies included in the systematic review (*n* = 172)

Study characteristic	Number of included studies (%)
**Year of publication**	
1990–1999	1 (1)
2000–2009	27 (16)
2010–2016	144 (84)
**Country**	
North America	124 (72)
Europe	29 (17)
Australasia	18 (10)
Other	1 (1)
**Study design**	
Non-original research	96 (56)
Editorial, letter-to-the-editor, website or news item	64 (37)
Literature review	29 (17)
Guideline	3 (2)
Original research	76 (44)
Cohort^a^	25 (15)
Quasi-experimental^b^	18 (10)
Qualitative	7 (4)
Mixed methods	6 (3)
Knowledge synthesis	3 (2)
Predictive modelling	3 (2)
Cross-sectional	3 (2)
Other^c^	11 (6)
**Focus of article**	
Identify low-value practices	86 (50)
Facilitate the de-implementation process	92 (53)
Evaluate de-implementation outcomes	82 (48)
Sustain de-implementation	18 (10)
**Targeted low-value practice**	
Therapeutic intervention(s)	42 (24)
Diagnostic intervention(s)	33 (19)
Both	15 (9)
Did not specify	82 (48)
**Reported barrier(s) to de-implementation**	138 (80)
**Reported facilitator(s) to de-implementation**	105 (61)

^a^Includes 8 studies where the cohort consisted of citations identified through electronic searches of the literature

^b^Includes interrupted time series, before-and-after, and non-randomized controlled trial

^c^Includes two consensus studies, two case reports, two surveys, two study protocols, one case-control study, one stakeholder engagement, one randomized clinical trial

Assessment of methodological quality was performed on original research articles only (Additional File 5). Most articles were of low-to-moderate quality. Median (inter-quartile range, IQR) scores reported as a percent of the maximum were 52% (45–57%) for quantitative studies, 45% (38–57%) for qualitative studies, and 46% (40–58%) for mixed-methods studies (Additional File 6).

#### Synthesized Determinants of De-implementation

From 437 barriers to de-implementation cited directly within included studies, our inductive synthesis yielded 29 distinct barriers, herein referred to as ‘barriers to de-implementation’. Among the barriers to de-implementation, those with most frequent representation within raw text included: lack of credible evidence defining a clinical practice as low-value (*n* = 90, 21%); entrenched norms and clinicians’ resistance to change (*n* = 43, 10%); patient demands and preferences (*n* = 28, 6%); challenges with stakeholder support (*n* = 27, 6%); perception of risk to patients associated with de-implementation (*n* = 13, 3%); and clinician challenges effectively communicating with patients about low-value practices (*n* = 5, 1%). From 280 facilitators, our inductive synthesis identified 24 distinct facilitators, herein referred to as ‘facilitators of de-implementation.’ Among the list of facilitators, those with most frequent representation within raw text included: stakeholder collaboration and communication (*n* = 43, 15%); availability of credible evidence (*n* = 33, 12%); physician-patient communication and shared decision-making about use of the targeted low-value practice (*n* = 24, 9%); audit and feedback regarding low-value practice use (*n* = 19, 7%); and clinical decision support tools (*n* = 13, 5%). The majority of barriers (*n* = 23/29, 79%) and facilitators to de-implementation (*n* = 17/24, 71%) derived from articles reporting original research. The ten most commonly cited determinants of de-implementation are presented in Additional File 7.

The determinants of de-implementation were mapped to relevant stakeholders (Table 2). With respect to barriers, 59% (*n* = 17/29) applied to clinicians, 55% (*n* = 16/29) applied to decision-makers, 38% (*n* = 11/29) applied to researchers, and 24% (*n* = 7/29) applied to patients. With respect to facilitators, 50% (*n* = 12/24) applied to clinicians, 50% (*n* = 12/24) applied to decision-makers, 38% (*n* = 9/24) applied to researchers, and 21% (*n* = 5/24) applied to patients. The full list of determinants mapped to stakeholders is available in Additional File 8.

**Table 2:** Ten most frequently cited determinants of de-implementation from the systematic review mapped to relevant stakeholders

Stakeholders	Frequently Cited Barriers	Frequently Cited Facilitators
Patients	• Patient demands and preferences •Challenges with stakeholder support	• Stakeholder involvement • Shared decision-making • Patient awareness of low-value care
Clinicians	• Lack of credible evidence • Disconnect between training and evidence • Fear of malpractice • Entrenched norms and clinicians’ resistance to change • Challenges with stakeholder support • Model of physician reimbursement	• Stakeholder involvement • Availability of credible evidence • Shared decision-making • Audit and feedback • Interactive clinician education • Clinical decision support
Decision-makers	• Lack of credible evidence • Lack of resources for de-adoption initiatives • Lack of data for identifying low-value care • Challenges with stakeholder support • Model of physician reimbursement • Lack of criteria for identifying low-value practices	• Stakeholder involvement • Availability of credible evidence • Cost-saving opportunity • Prioritized low-value practices • Established and credible assessment criteria to identify low-value care
Researchers	• Lack of credible evidence • Lack of resources for de-adoption initiatives • Lack of data for identifying low-value care • Challenges with stakeholder support • Lack of criteria for identifying low-value practices	• Stakeholder involvement • Availability of credible evidence • Prioritized low-value practices • Established and credible assessment criteria to identify low-value care

#### Mapping Determinants of De-implementation to the Theoretical Domains Framework (TDF)

Additional File 9 presents the complete list and counts of determinants of de-implementation mapped to the most relevant domain of the TDF. The TDF domain with the greatest number of mapped unique determinants was ‘Environmental context and resources’, which pertains to the circumstances of the clinician’s situation or environment that influences behavior change. Relevant barriers within this domain included lack of resources for de-implementation initiatives and a healthcare system that is complex and unconducive to change. Relevant facilitators within this domain include positive influence from political or industry stakeholders and the perception of a cost-savings opportunity. Many determinants also mapped to the ‘Knowledge’ domain within the TDF. Here, barriers pertained to the identification or awareness of low-value practices, such as lack of evaluation methods and data for identifying candidate low-value practices and lack of criteria for identifying low-value practices. Facilitators within the Knowledge domain included interactive clinician education about the targeted low-value practice and/or de-implementation and having prioritized low-value practices.

### Stakeholders Interviews

Physicians (*n* = 6, 35.3%), nurses (*n* = 6, 35.3%), and decision makers (*n* = 5, 29.4%) were represented in the stakeholder interviews, of which nine (52.9%) were female (Table 3). Years of experience in critical care medicine was most commonly 6–10 years (*n* = 9, 52.9%).

**Table 3:** Interview participant characteristics

Characteristic	Number of participants (***n*** = 17)
**Role**	
Physician	6
Nurse	6
Decision Maker	5
**Sex**	
Female	9
Male	8
**Year of Birth**	
1950–1959	1
1960–1969	1
1970–1979	6
1980–1989	7
1990–1999	2
**Years working in critical care medicine**	
1–5	4
6–10	9
10+	4

#### Synthesized Determinants of De-implementation

Of the 29 distinct barriers and 24 distinct facilitators identified in the systematic review, 23 (79%) barriers and 20 (83%) facilitators were independently identified by stakeholders as influencing de-implementation in critical care medicine. The full list of determinants identified in stakeholder interviews with exemplar quotations is available in Additional File 10. The barriers developed in the systematic review that were not expressed in stakeholder interviews included: concern with response from insurance companies, lack of clinical decision support, lack of criteria for identifying candidate low-value practices, lack of understanding of barriers & facilitators, time constraints during patient visits, and unclear goal for de-implementation. Facilitators not expressed in Phase II included: infrastructure for accurately measuring the use of low-value practices, performance incentives, prioritized low-value practices, and value-based insurance design.

#### Determinants Mapped to Conceptual Model for Facilitating De-implementation

**Fig. 1:**
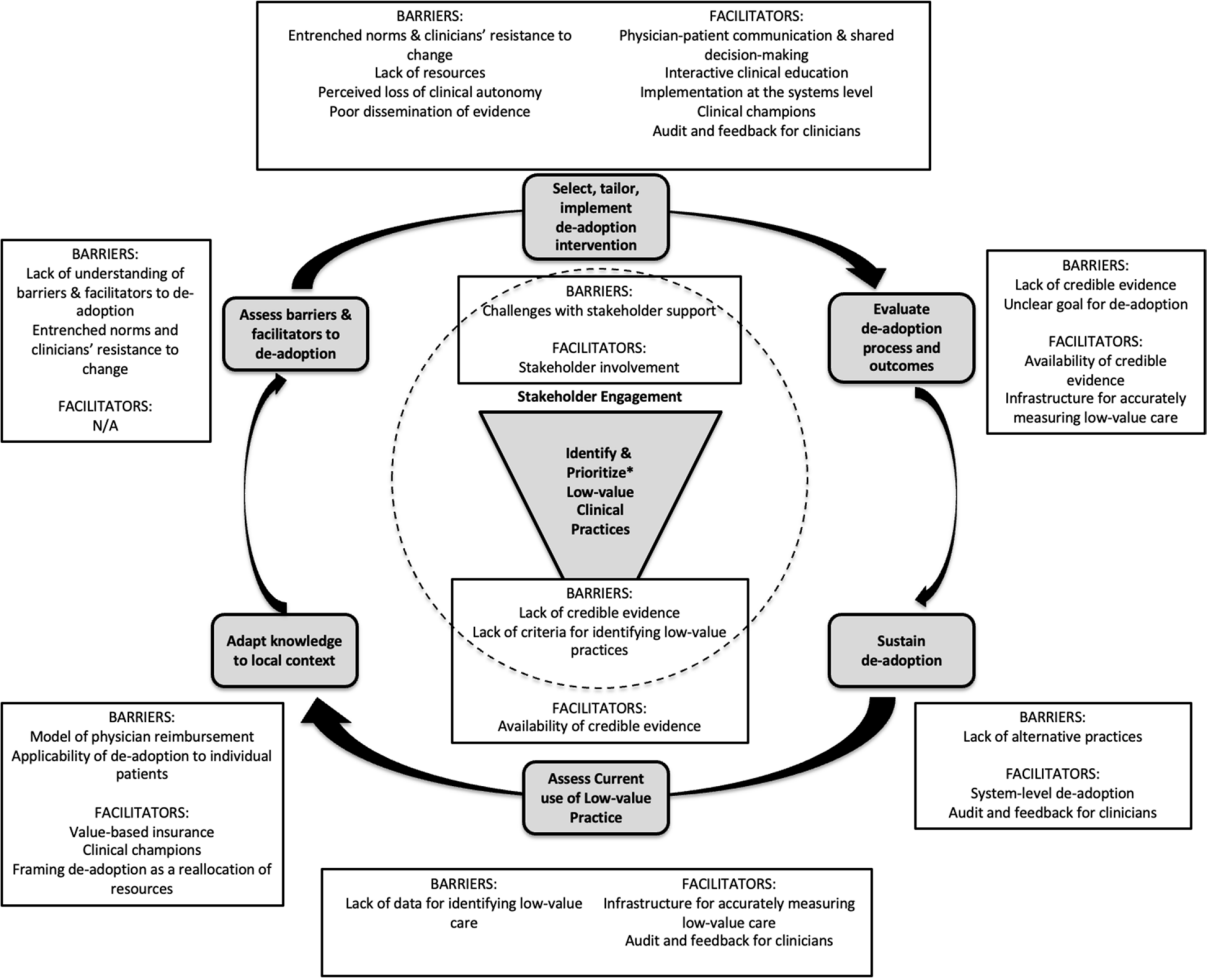
Most frequent barriers and facilitators to de-implementation identified in the systematic review and stakeholder interviews mapped to the Conceptual Model for Facilitating De-Implementation [15]

Figure 1 depicts the most frequently cited barriers and facilitators to de-implementation identified in the systematic review and interviews mapped to the conceptual model for facilitating de-Implementation. While similar determinants were found to act as barriers and facilitators to de-implementation in both phases of this study, a few differences emerged. The systematic review identified systems-level determinants such as value-based insurance and physician models of reimbursement as well as the importance of having available infrastructure for measuring low-value care that did not emerge during the stakeholder interviews. Interviewees emphasized factors more specific to the clinical context such as the importance of objectively identifying candidate low-value practices and the importance of incorporating audit and feedback into any de-implementation intervention.

## Discussion

In this study, we employed several methodologies to develop and a comprehensive list of the determinants of de-implementation of low-value care from the published literature and to compare these to determinants described by those with lived experience with de-implementation within critical care medicine. From 172 articles, the systematic review with conventional content analysis identified 29 distinct barriers and 24 facilitators, of which semi-structured interviews independently cited approximately 80% of identified barriers and facilitators as determinants of de-implementation in critical care medicine. To our knowledge this is the first multi-method study to develop and corroborate a list of determinants of de-implementation. Although corroboration was confined to stakeholders from one medical discipline, the breadth of the literature from which the determinants were synthesized, combined with the similarities between the identified determinants compared to previous literature, and their otherwise actionable nature (e.g., stakeholder engagement), suggest applicability outside the test discipline, and an opportunity to influence de-implementation efforts more broadly. Collectively, the findings from this study may help to explain the observed effects of prior de-implementation interventions and inform the development of future initiatives that aim to de-implement low-value care.

Similar to prior studies examining barriers and facilitators to evidence use [39–41], the current study underscores the powerful influence of the availability and credibility of scientific evidence on de-implementation. Cabana’s seminal work examining why physicians don’t follow clinical practice guidelines found that lack of awareness, familiarity or agreement with a guideline accounted for nearly 40% of identified barriers and three of seven major barrier categories [39]. In a prior systematic review of barriers and facilitators to gaps between evidence and clinical practice, Cochrane and colleagues identified a lack of utility of evidence, in addition to lack of its awareness, as top barriers to behavior change [41]. More recently, Tricco and colleagues examined barriers and facilitators to uptake of systematic reviews by healthcare managers and decision-makers [40]. They found that lack of awareness of a systematic review, lack of agreement with systematic review methods in general, and lack of agreement with results of specific systematic reviews were the main determinants of systematic review utilization among managers and decision-makers. More specific to de-implementation, two recent studies examined determinants of use and de-implementation of low-value care [24, 25]. van Dulmen et al. found a predominance of barriers related to individual healthcare provider and patient attitude and knowledge as well as availability of resources within an organization [25]. Many articles cited patient preference and expectations combined with physicians’ communication and time as important barriers to de-implementation. Augustsson et al. also identified that patient expectations and physicians’ fear of malpractice are prominent determinants of de-implementation [24]. Our study similarly identified patients’ knowledge and expectations and clinicians’ resistance to change as frequent determinants of de-implementation of low-value care. However, in our study, cited more frequently within the included articles were the importance of the quality and availability of evidence that underpins a de-implementation initiative as well as stakeholder collaboration. This was subsequently identified by interviews among stakeholders with lived experience with de-implementation. Taken together with these two prior studies, there is now a more advanced understanding of the determinants of de-implementation, how they are similar to implementation, and the nuanced differences. Future de-implementation initiatives should focus on clinical practices defined as low-value by strong scientific evidence, seek early stakeholder engagement including patients, healthcare professionals, and decision-makers, and broadly educate stakeholders regarding the risks and benefits of de-implementing clinical practices deemed to be low-value.

That a frequent barrier to de-implementation is a lack of credible evidence demonstrating a given clinical practice to be low-value helps explain why after nearly a decade speaking about low-value care [42], consensus has yet to be reached on what constitutes low-value care, and how it should be identified [14]. Naturally occurring clinical heterogeneity creates a spectrum of value within clinical practice; a test or treatment may be considered low-value in one clinical context but not in another, and it is difficult for science to adequately examine efficacy or effectiveness of all clinical practices in all contexts. In contrast to prior reviews examining determinants of evidence use [39–41], methods for identifying clinical practices that are low-value and prioritizing them for de-implementation was more commonly cited as a barrier to de-implementation compared to implementation and appears critically important to stakeholder receptiveness to de-implementation initiatives. It is thus clear that defining methods for identifying and prioritizing low-value clinical practices for de-implementation should be a priority within de-implementation research. This process needs to be systematic, grounded in evidence and contemporary data demonstrating overuse of the low-value practice, and from the beginning engage relevant stakeholder groups, and not simply be the distillation of experts hand-picking from available literature, or their own personal lists [15].

Our findings also demonstrate that framing can impact the effectiveness of de-implementation efforts. Framing de-implementation as an opportunity for cost-savings or reallocation of resources was identified as a facilitator in the systematic review, whereas framing it as cost-cutting was identified as a barrier. These slight but important differences in framing can substantially impact stakeholder attitudes towards behavior change. Patient demands and preferences were also identified as a common barrier to de-implementation in the systematic review. This suggests that even if clinicians intend to change their behavior and reduce low-value care, their intentions could be derailed by patient preferences for low-value tests (e.g. MRI for low-back pain [43]) or treatments (e.g. antibiotics for viral infections [43]), and greater attention to the importance of patient-engagement in de-implementation interventions is needed [44].

The results of this study need to be interpreted in the context of its limitations. First, the search strategy restrictions by date and language may have caused omission of relevant articles. However, the 172 included articles as well as 437 raw text barriers and 280 raw text facilitators that were synthesized into 29 and 24 unique barriers and facilitators, respectively, are larger in number than that described in prior reviews on determinants of evidence use [39–41], and describe concepts congruent with the main results of those reviews. Furthermore, the search was conducted in 2016, and articles published since this time will not have been included. However, recent evidence syntheses by Augustsson et al. and van Dulmen et al., found similar challenges facing de-implementation initiatives [24, 25], suggesting potentially missed citations are unlikely to change our main results. Second, synthesizing a list of distinct barriers and facilitators from individual articles was a potentially subjective process. To mitigate this challenge, we had two reviewers code and review emerging representative barriers and facilitators. Third, is the nature of the included studies. A number of included citations were non-original research (only one randomized clinical trial), and the majority of original research citations were of low-to-moderate methodological quality from high-income countries. Therefore, the list of distinct barriers and facilitators derives from a cohort of mostly low methodological quality articles specific to the high-income country context. Despite this, the majority of the distinct barriers and facilitators were derived from data extracted from original research articles, and the final list is a comprehensive representation of what is reported in the literature. Fourth, the specific nature of our interview sample (critical care medicine stakeholders from one province) may limit transferability of the findings to other stakeholder groups. However, the high level of agreement between the interviews and the systematic review suggest that interviews exploring determinants of de-implementation in other medical disciplines are likely to yield similar results. Lastly, while there was an extended time period between conducting the search for the systematic review and conducting stakeholder interviews, the fact that determinants identified from the literature overlapped with determinants noted by stakeholders suggests that the extended timeframe between phases is unlikely to have influenced the main results.

## Conclusions

Using a multi-method approach, this study identified 29 distinct barriers and 24 distinct facilitators to the de-implementation of low-value care from the published literature, of which the majority were also cited in interviews among stakeholders with lived experience with de-implementation in critical care medicine. Lack of credible evidence defining a practice as low-value, entrenched norms and clinicians’ resistance to change, and challenges with securing, mobilizing and maintaining stakeholder support were identified as frequent barriers, while stakeholder collaboration and communication, availability of credible evidence, and execution of de-implementation at the system-level were the most frequent facilitators. Additional work is required to determine if the identified list of determinants to de-implementation is relevant to stakeholders working in other medical disciplines, and to develop a comprehensive, evidence-informed model for de-implementation. However, in the meantime, de-implementation determinants identified in this study may be used to inform future de-implementation initiatives.

## Supplementary Information

**Additional file 1.** MEDLINE Search Strategy.

**Additional file 2.** Interview Guide.

**Additional file 3.** Selection of articles included in the review.

**Additional file 4 **Bibliographic table of included studies (*n* = 172).

**Additional file 5. **Quality assessments of included original research articles (*n *= 74)

**Additional file 6.** Summary of QATSDD scores for included original research articles.

**Additional file 7.** Ten most commonly cited determinants of the de-implementation of low-value practices.^a^ A: barriers, B: facilitators. ^a^Increased size and depth of colour indicate a higher number of citations underpinning the reported barrier or facilitator.

**Additional file 8.** Determinants of the de-implementation of low-value practices mapped to relevant stakeholders.

**Additional file 9.** Determinants of the de-implementation of low-value practices mapped to the Theoretical Domains Framework (TDF).

**Additional file 10.** Determinants identified in stakeholder interviews with exemplar quotations.

## Data Availability

The datasets generated during the current study are available from the corresponding author upon reasonable request.

## Abbreviations

PRISMA: Preferred Reporting Items for Systematic Reviews and Meta-Analyses
CENTRAL: Cochrane Central Register of Controlled Trials
QATSDD: Quality Assessment Tool for Studies with Diverse Designs
ICUs: Intensive Care Units
TDF: Theoretical Domains Framework
