# Supplementary material for: Determinants of the de-implementation of low-value care: a multi-method study
Source: BMC Health Serv Res. 2022 Apr 6;22:450. doi: 10.1186/s12913-022-07827-4 (PMC8985316; doi:10.1186/s12913-022-07827-4)
Supplement: Supplementary file 1 — Additional file 1. MEDLINE Search Strategy. [file 12913_2022_7827_MOESM1_ESM.docx]

**Additional File 1. MEDLINE Search Strategy**

| **Line** | **Search terms** |
| --- | --- |
| 1 | ((abandon* or contradict* or refute* or refuting or reassess* or re-assess* or obsole* or revers* or delist* or de-list* or disinvest* or dis-invest* or discontinu* or dis-continu* or decommission* or de-commission* or deadopt* or de-adopt* or de-implement* or deimplement* or "health care' withdraw*" or (no adj benefit*)) adj5 (healthcare or technolog* or device* or intervention* or health practi?e* or medical or medical practi?e* or procedur* or drug or drugs or biotechnology*)).tw. |
| 2 | limit 1 to English language |
| 3 | limit 2 to animals |
| 4 | Limit 2 to (animals and humans) |
| 5 | 3 not 4 |
| 6 | 2 not 5 |
| 7 | Limit 6 to (“all infant (birth to 23 months)” or “newborn infant (birth to 1 month)” or “infant (1 to 23 months)” or “preschool child (2 to 5 years)” or “child (6 to 12 years)”) |
| 8 | 6 not 7 |
| 9 | Limit 8 to yr=”1990-Current” |
