# Supplementary material for: Determinants of the de-implementation of low-value care: a multi-method study
Source: BMC Health Serv Res. 2022 Apr 6;22:450. doi: 10.1186/s12913-022-07827-4 (PMC8985316; doi:10.1186/s12913-022-07827-4)
Supplement: Supplementary file 2 — Additional file 2. Interview Guide. [file 12913_2022_7827_MOESM2_ESM.docx]

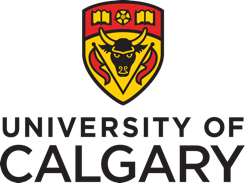
**Study Title:** De-Adopting Low Value Practices in Critical Care Medicine

**Sponsor:** Canadian Institutes of Health Research

**Principal Investigator:** Dr. Jeanna Parsons Leigh

**Co-Investigators:** Drs. Kirsten Fiest, Daniel Niven, Thomas Stelfox

**Preamble – Key Points:**

- Overview of study
  - We are conducting this study to learn more about the process of removing low-value practices in critical care medicine.
  - Low value care practices consists of medical tests and treatments that are unnecessary, potentially harmful, or not cost-effective.
  - The removal or reduction of low value care is sometimes called de-adoption.
- We wanted to interview you as you work in the ICU/ critical care setting.
  - We want to understand the barriers and facilitators that you think may contribute to the success or failure of removing low value practices.
  - We also want to know about contextual factors.
  - Interview guide
  - The interview will take ~30-45min
  - We will also ask you a few brief demographic questions at the start, then we will move into the main interview questions.
  - If you have any additional thoughts related to this study that aren’t addressed with the questions, we really want to hear these, so please include them as we go.
  - At the end of the interview, as we are in the pilot phase of this study, we will also ask for your feedback on the interview process.
- Consent
  - Did you have time to read the consent form?
  - This interview is completely voluntary.
  - If you want to skip a question or end the interview, that is completely fine, just let us know.
- Confidentiality
  - All information you share will be ‘de-identified’, meaning your name will not appear anywhere in the written transcripts or in any reports we produce.
  - No personal information will be shared with anyone outside of our study team.
  - We hope you will be okay if we record this interview.
    - This is so we don’t lose important details from our discussion. Only our study team and transcriptionist will hear this.

Do you have any questions about the study before we get started?

***Section 1: Structured Demographic Questions***

Your demographic information and contact info will be stored in a password protected database that is only accessible to the study research team. If you are not comfortable answering any of the below questions you are welcome to skip any or all of those you do not wish to answer.

1. What is your role in your organization? How long have you worked in your current role?

__________________________________________________________________________________

2. How many years have you worked in critical care medicine?

__________________________________________________________________________________

3. a) *[MD’s Only]* What is your base specialization?

__________________________________________________________________________________

b) *[MD’s and RN’s]* How many years have you been in practice (*for Fellows*: what Postgraduate Year)?

__________________________________________________________________________________

c) *[All]* What is the highest level of post-secondary education you’ve attained (excluding medical school)?

⃝ Diploma or certificate ⃝ Bachelor ⃝ Master ⃝ PhD

⃝ Prefer Not to Disclose

4. What type of institution are you currently working in (academic vs. non-teaching, regional vs. urban, etc.)?

__________________________________________________________________________________

5. What is your sex?

⃝ Male ⃝ Female ⃝ Prefer Not to Disclose

In what year were you born?______________________

***Formal Consent***

I will now turn on the audio recorder and ask you a few questions to formally record your consent before moving onto the main questions of the interview.

****Turn on the digital recorder****

1. Are you familiar with all of the study details?
2. Have all of your questions been adequately answered?
3. Do you agree to be interviewed for research purposes?
4. Do you have any questions or concerns about the process? If not, let’s begin….

***Section 2: Semi-Structured Questions***

**Familiarity with ‘de-adoption’**

1. How familiar are you with initiatives or programs designed to address the reduction or removal of low-value care practices in healthcare? (ie. removal/ reduction of the standard do/don’t do’s in your ICU)
   1. For example, have you heard of Choosing Wisely? (an international campaign designed to help clinicians and patients engage in conversations about unnecessary tests, treatments and practices)

**Critical care setting**

1. This study is focused on the removal or reduction of low-value care specifically in critical care settings. In comparison to other healthcare settings, are there any unique considerations to critical care as it relates to the removal of low-value practices?

**Example of a de-adoption effort**

1. Can you describe an example of a low-value practice that was removed in your ICU? (for example, removal of a procedural therapy or reduction in use of a particular medication over time)
   1. As it relates to your example above, were there any interventions or strategies used or considered to support the removal or reduction of this practice? of the harmful/ineffective practice?
   2. How successful was it? How did you measure success? Was it sustainable? Why?

**Barriers or challenges to de-adoption**

1. In your opinion, what are some of the reasons healthcare providers would still use a practice that has been identified as being of a low-value? (*Note for interviewer: Examples include routine chest radiographs or the routine transfusion of red blood cells)*
2. How do you perceive contextual factors (i.e. sex/age/province/base specialty) influencing potential removal or reduction efforts?
   1. How does being situated in an academic/ non-academic hospital setting influence removal/ reduction efforts in your ICU?

**De-adoption processes**

**Thinking specifically of your unit/ICU and how they identify and then remove low-value practices:**

1. How are clinical practices *identified* as low-value?
   1. Who are the key players involved with identifying low-value practices?
   2. Are patient and family partners or members of the public involved in this process?
   3. Should they be?
   4. Are there any other stakeholders involved in the identification phase?
2. How are de-adoption efforts *implemented*?
3. Who are the key players in developing and driving de-adoption efforts/ intervention?
4. Are patient and family partners or members of the public involved in this process?
   1. Should they be?
5. Are there any other stakeholders involved in the implementation phase?
6. What are the main facilitators to consider with the removal of a low-value practice in your ICU?
7. What are the main barriers ICUs face in effectively coordinating removal efforts between different stakeholders?
8. Is there anything else that we haven’t had the opportunity to talk about today that you would like to add?
9. Is there anyone else you can think of who we should contact for an interview on this topic?

Thank you for sharing your opinions and experiences with me. If you have no further insights to share, I will now turn off the recorder.
