# Supplementary material for: Determinants of the de-implementation of low-value care: a multi-method study
Source: BMC Health Serv Res. 2022 Apr 6;22:450. doi: 10.1186/s12913-022-07827-4 (PMC8985316; doi:10.1186/s12913-022-07827-4)
Supplement: Supplementary file 3 — Additional file 3. Selection of articles included in the review. [file 12913_2022_7827_MOESM3_ESM.docx]

**Additional File 3. Selection of articles included in the review**

54,169 Citations identified through electronic database searches

EMBASE 22,013

MEDLINE 15,115

Cochrane 11,079

CINAHL 5,962

256 Citations identified through other sources

Duplicates removed
(n = 19,057)

Unique citations after duplicates removed
(n = 35,368)

Excluded
(n = 35,031)

Full-text articles excluded (n = 216)

No discussion of barriers/facilitators = 102

Primary evidence for reversal = 27

Neither adoption nor

de-adoption = 35

Adoption focus = 32

Other = 20

Full-text articles assessed for eligibility
(n = 337)

Citations identified from included reference lists, personal library, and KT experts
(n = 51)

Articles included in the systematic review

(n=172)
