# Supplementary material for: Determinants of the de-implementation of low-value care: a multi-method study
Source: BMC Health Serv Res. 2022 Apr 6;22:450. doi: 10.1186/s12913-022-07827-4 (PMC8985316; doi:10.1186/s12913-022-07827-4)
Supplement: Supplementary file 4 — Additional file 4 Bibliographic table of included studies (n = 172). [file 12913_2022_7827_MOESM4_ESM.docx]

**Additional File 4. Bibliographic table of included studies (n=172)**

| Author, year | Study design | Reported barriers | Reported facilitators |
| --- | --- | --- | --- |
| Admon(1), 2014 | Editorial/letter-to-the-editor | - Knowledge of physicians with respect to Choosing Wisely guidelines - Physicians awareness of guidelines - Physicians may not agree with guidelines (ex. Guidelines do not seem trustworthy because they were not developed from a rigorous review of evidence) - Guidelines too complex - Physicians' perception that they are not responsible for controlling the costs of health care | - Engaging consumer and patient advocacy groups - Improved share decision making - Payment system based on outcomes - Incorporating guidelines into the electronic medical record - Increase physician awareness of the costs associated with healthcare |
| Agiro(2), 2016 | Editorial/letter-to-the-editor | - Changes in the payment system is not enough | - Targeting delivery systems because they are linked to clinicians with close patient contact - Data feedback to clinicians and systems interventions (ex. Decision support in electronic records) - Patient education |
| Angus(3), 2015 | Editorial/letter-to-the-editor | - Difficulty identifying when a low-value practice is effective and when it's not - Physicians' belief that they can use the low-value practice effectively | - Engaging experts and physicians |
| Archbald-Pannone(4), 2016 | Editorial/letter-to-the-editor | - Wrongly applying evidence from one cohort to the next (ie. Not all evidence is generalizable) | - None reported |
| Arseneau(5), 2016 | Website, news item, professional society document | - None reported | - Getting doctors to examine their own practices |
| Atwater(6), 2009 | Before-and-after | - None reported | - Presentation at major biomedical meetings - Publication in well-respected journals |
| Azermai(7), 2014 | Mixed methods | - Concern about patient's quality of life - Risk of harm after discontinuation of an established practice | - None reported |
| Balekian(8), 2016 | Cohort | - Lack of general guidelines that all institutions can follow | - None reported |
| Banta(9), 1990 | Narrative review | - Resistance due to established clinical training - Lack of funds and resources to assess technology | - Public health surveillance of technologies |
| Bauman(10), 2016 | Editorial/letter-to-the-editor | - Potential limits on applicability of guidelines | - None reported |
| Beller(11), 2012 | Editorial/letter-to-the-editor | - Objectively determining clinical appropriateness of treatments with consideration of safety and cost effectiveness | - Educating clinicians about ordering tests |
| Bernstein(12), 2015 | Website, news item, professional society document | - Lack of evidence - Lack of alternative treatments to the low-value practice - Current payment system (ex. Fee for service) - Need to consider outcomes of patients over other things (ex. Money) - Personal interests (ex. Financial greed) | - Patient's knowledge (ex. Knowing some information about a procedure before seeing a specialist) - Technology development to make information more available - Education and educational tools (ex. Apps) for patients |
| Bhatia(13), 2015 | Narrative review | - Malpractice concerns - Patient preference and demand - Lack of automated decision support - Payment policies - Lack of research on low-value practices and their utilization rates - Need to consider outcomes of patients | - Decision support tools - Patient and provider feedback - Studying how patients and physicians are reacting to change, which helps identify areas of improvement - Development of tools to measure utilization rates, stakeholder attitudes, etc. |
| Blumenthal-Barby(14), 2013 | Narrative review | - Habits and experience of physicians - Difficulty defining low-value care and describing what benefits look like - There are many ways to implement change, but not all the methods studied help change behaviours | - None reported |
| Borasio(15), 2016 | Narrative review | - Fear of lawsuits - Lack of evidence - Lack of awareness of the proper decision making process - When a patient is about to pass away, doctors may do more than necessary | - Shared decision making between doctor and patient - Clear communication between physician and patient |
| Brunt(16), 2003 | Interrupted time series | - Media communication of medical research | - Pressure from patients - Clinicians having access to detailed information regarding treatments |
| Callaghan(17), 2015 | Website, news item, professional society document | - Lack of research done on interventions known to reduce overutilization - Lack of data regarding what the cost of certain practices are | - None reported |
| Casarett(18), 2016 | Editorial/letter-to-the-editor | - Tendency to overestimate the effects of one's actions - Only looking at evidence of impact (ex. Interventions that show improvements in patient's health) - Lack of research into how "number sense" (ex. Being able to estimate how effective a treatment is) can be incorporated into medical education | - None reported |
| Clement(19), 2015 | Editorial/letter-to-the-editor | - Lack of methods to evaluate whether or not low-value practice usage has decreased - Physicians feel uncertain because there are too many guidelines to follow | - Rigorous implementation and evaluation of changes implemented - Financial incentives - Feedback and performance reporting - Using dashboards |
| Colla(20), 2016 | Cross-sectional study | - Patient demand for services - Lack of financial incentives - Fear of malpractice - Physicians feel that the uncertainty involved in patient care is disconcerting - Lack of understanding of costs of tests and procedures to the health care system | - More decision-support tools - Education physicians and patients about low-value practices - Shared decision-making |
| Colla(21), 2015 | Cohort study | - Malpractice liability concerns - Fear of misdiagnosing a patient - Patient demand for services | - Identification of low-value services - Engaging/educating physicians and patients - Physician incentives |
| Colla(22), 2015 | Cohort study | - Identifying low-value services - Not defining what constitutes appropriate or wasteful care - Quantifying the impact of low-value practices | - Educating care providers |
| Cooper(23), 2010 | Editorial/letter-to-the-editor | - Lack of resources to support policies - Lack of agreement about comparative cost-effectiveness within and between disciplines - Lack of research and evidence - Disputes over evidence - Political, clinical, and social resistance to disinvestment - Perception that the term 'disinvestment' is problematic | - Better evidence based clinical decision making - Better integration of the healthcare system with community care, social care, primary/secondary providers, etc. - Promoting a culture of collaboration - Greater dialogue to promote change |
| Crosby(24), 2016 | Editorial/letter-to-the-editor | - Pressure to keep patients happy - Pressure to make quick and efficient decisions | - None reported |
| Davidoff(25), 2015 | Editorial/letter-to-the-editor | - Preference for familiar treatments, even if these treatments are harmful - Loss of revenue if certain services are abandoned - Tendency to follow experiments with positive results - Patient expectations - Peer pressure from colleagues | - Developing new ways to determine the effectiveness of a treatment |
| de Boer(26), 2013 | Editorial/letter-to-the-editor | - Fear of litigation and malpractice - Some procedures performed may be money driven - Procedures are ordered to meet obligatory standards - Patient demands | - None reported |
| Deyell(27), 2011 | Interrupted time series | - Preference of positive clinical trials over negative ones - Lack of agreement on data interpretation - Contradiction of long-held beliefs - Patient expectations | - None reported |
| Donaldson(28), 2010 | Editorial/letter-to-the-editor | - Healthcare system structure (ex. Budget increases led to managers desensitized to managing scarce resources) - Change requires time and effort - Need to involve multiple stakeholders - Lack of consensus as to how outcomes are measured | - None reported |
| Dorsey(29), 2013 | Editorial/letter-to-the-editor | - Recommendations are overwhelmingly negative (ex. If the guidelines tell you what not to do, then you won't know what you should do; this may create the impression that cost-containment is more important than patient health outcomes) - Procedures are performed purely for reimbursement rather than medical necessity - Physicians lack political support to create change | - Changes in reimbursement (the authors argued that since public awareness is not likely to change behaviours, changing the financial structure may be the better solution) - Consumers can help change the incentives that are affecting their care |
| Edwards(30), 2014 | Predictive/economic modelling | - Financial and budget concerns (ex. lack of funding) - Support from local public health groups - Lack of evidence showing the impact of targeting certain practices | - Early intervention - Prioritizing and identifying which practices to target - Promoting an evidence based culture |
| Elshaug(31), 2008 | Qualitative | - Lack of evidence - Lack of resources to support research advancement - Resistance to change due to established training | - Convince policy makers that proposed changes are necessary |
|  |  |  |  |
| Elshaug(32), 2007 | Narrative review | - Lack of reliable ways to identify and prioritize technologies and practices - Lack of resources to support disinvestment - Lack of evidence to support disinvestment (ex. Showing a particular technology is ineffective) - Lack of resources and funds to support a research agenda - Overwhelming evidence (ex. Having to analyze a 700 page document) - Resistance due to established clinical training - Satisfying the interests of multiple groups - Demands of consumers and suppliers - Fear that disinvestment infringes upon personal freedom and autonomy | - Partnership between government, colleges, and relevant stakeholders - Establishing goals within a committee (ex. Step 1 - identify technologies, Step 2 - Assess potential candidates for disinvestment, etc.) - Providing more funding to build services and agendas with a focus on disinvestment - Conduct assessments of potential obsolete and ineffective treatments |
| Elshaug(33), 2009 | Website, news item, professional society document | - A decentralized system (ex. Federal and provincial) needs more coordination in order to bring about change - Lack of financial resources - Lack of ability to identify and prioritize ineffective technology and services - Political, clinical, and social support from key stakeholders or groups - Lack of evidence | - Developing new methods to evaluate technology (ex. New evidence, geographic considerations, patient experience, etc.) - Cooperation between government, professional colleges, stakeholder groups, and citizens to foster a committed effort towards de-implementation. - Sufficient resources to support plans - A central administration to enforce rules, regulations, and the process of de-implementation |
| Elshaug(34), 2009 | Narrative review | - Developing a systematic method to review and assess practices - Not wanting to make difficult choices in the context of scarcity - Determining who will fund, oversee, assess, make decisions, and implement disinvestment policies | - Identifying ineffective practices - Jointly funded program by all relevant stakeholders |
| Elshaug(35), 2013 | Editorial/letter-to-the-editor | - Identifying and prioritizing disinvestment candidates - Health care structure (ex. Pay for performance) - Variations in the effectiveness of a drug (ex. Drug X benefits one group, but harms another group) - Lack of data and evidence makes it hard to assess low-value practices and implement change - Creating a system to determine who pays what and monitoring the effectiveness of the system | - Incentives to promote disinvestment - Rigorous methods to collect and assess data |
| Ferguson(36), 2012 | Narrative review | - Finding a balance between financial stewardship and duty to patients | - Greater communication between doctor and patient regarding treatments |
| Ferrari(37), 2015 | Non-randomized controlled trial | - Fear of misdiagnosing a patient | - None reported |
| Ferrari(38), 2016 | Narrative review | - Education and awareness is not enough to change behaviours | - Building implementation tools into the system (ex. Indicating how frequent to order a test on a requisition form) |
| Fessele(39), 2016 | Guideline | - None reported | - Communication with patients - Educating patients about low-value practices |
| Fleisher(40), 2014 | Editorial/letter-to-the-editor | - None reported | - Better communication between physician and patient |
| Fritzler(41), 2016 | Narrative review | - Patient demands - Lack of analysis regarding the costs or savings if changes are made - Concern regarding health outcomes when implementing change - Reliance on evidence that is old - The Choosing wisely recommendations themselves may have problems - Lack of understanding of why a particular treatment is used and when it should be used | - Greater communication between doctor and patient regarding treatments |
| Garner(42), 2012 | Other | - Lack of evidence - Data on usage difficult to obtain | - Identifying and evaluate evidence and treatments - Stakeholder engagement - Strong leadership and political support - Developing a process for monitoring and communicating - Incentives to promote disinvestment |
| Garner(43), 2011 | Editorial/letter-to-the-editor | - Lack of national data to support a resource saving model - Stakeholder interests and perspectives - Lack of evidence that shows how effective a treatment is | - Creating a database - Political and professional support - Consulting stakeholders |
| Gerdvilaite(44), 2011 | Systematic evidence synthesis | - Lack of criteria to assess obsolete technology - Lack of large scale disinvestment projects - Local conditions and interests of multiple groups - Lack of funding and resources | - Withdrawing reimbursement - Consistently reminding healthcare professionals - Creating a database |
| Gershengorn(45), 2013 | Cohort | - Willingness of healthcare professionals to perform a certain procedure - Performing procedures on patients with certain characteristics | - None reported |
| Gidwani(46), 2016 | Cohort | - Lack of financial incentives - Fear of malpractice | - Care provider and patient education |
| Glauser(47), 2014 | Website, news item, professional society document | - Patient demand for certain services - Financial barriers or regulations | - Patient education - Stakeholder engagement |
| Gnjidic(48), 2015 | Editorial/letter-to-the-editor | - Resistance to a potential loss function - Burden of evidence requirements - Lack of ways to encourage optimal use - Lack of balance between identifying low value practices and being able to evaluate them | - Standardizing the definition of de-implementation |
| Greene(49), 2015 | Case report/series | - Lack of resources - Fear of litigation - Patient expectations - Medical liability | - None reported |
| Haas(50), 2004 | Interrupted time series | - Skeptic about new results | - Publication of data and evidence |
| Hass(51), 2012 | Narrative review | - Generally, if treatments and technologies are regarded as safe (ie. Not shown to do harm), they are not reviewed - Tendency for guidelines to emphasize what should be done over what shouldn't be done - Some technologies may be ineffective in one area, but effective in another - Difficulty identifying candidates for disinvestment - Lack of resources for research - Clinicians and consumers fear they will lose something, but may not see that something can be gained from disinvestment | - Using incentive (ex. Pay for performance) |
| Haines(52), 2014 | Narrative review | - Lack of uncertainty regarding evidence related to effectiveness of treatments - Pressure from budget cuts and competition for resources may force decision makers to make decisions without considering evidence - Difficulty conducting research (ex. Randomized controlled trials) due to fear of harm to patients | - Designing new experimental set-ups to determine effectiveness of treatments - Evaluating clinical studies more frequently to ensure patient safety alleviates stakeholder concerns - Changing the perception that disinvestment is not about "removing resources," but more about "reallocating" resources |
| Halpern(53), 2014 | Website, news item, professional society document | - Evidence itself may not induce change in practice - Specialities only care about practices in their field, and they have no regard for other specialities - Choosing Wisely recommendations may only target the individual-level, but other methods will be needed if targeting the system level (ex. Staffing patterns) - If reimbursement structures are modified, physicians need to be made aware why its modified and what is expected of them | - Having strong evidence - Greater communication between doctor and patient regarding treatments |
| Harris(54), 2013 | Website, news item, professional society document | - Poor quality evidence - Lack of financial, political, and social support - Structure of the health care system (ex. Fee for service funding models, etc.) - Difficulty identifying treatments that can be dis-invested - Satisfying the interests of multiple groups - Clinician's resistance to change - Lack of communication between different agencies - Lack of surveillance after implementation of policies - Not continuously evaluating the effectiveness of technologies and practices - Desire to do something instead of nothing when it comes to treatments - Fear of patient litigation for not doing anything - Clinicians medical training may interfere with how they respond to evidence that goes against what they have learned | - Stakeholder engagement and agreement - Good quality evidence, especially if safety issues are discussed - Addressing not only the evidence available, but the people involved - Dis-investment and investment in new technologies need to happen simultaneously to avoid budget strains - Incentives to promote disinvestment - Awareness of the benefits and drawbacks of treatments (ex. Guidelines, etc.) |
| Harvey(55), 2014 | Editorial/letter-to-the-editor | - Lack of national data management - Tendency for specialties to point fingers at other specialties, but not examine their own practices - Government interference | - None reported |
| Hauptman(56), 2006 | Interrupted time series | - None reported | - New findings and evidence |
| Hawasli(57), 2015 | Guideline | - Fear of misdiagnosing a patient - Malpractice liability - Patient preferences and demands - Lack of validation of clinical guidelines | - None reported |
| Henshall(58), 2012 | Qualitative | - Perception of a disadvantage when removing an existing technology - Patients’ sense of entitlement to treatments that are traditionally available - Heterogeneous patient outcomes - Clinician knowledge and training - Lack of evidence supporting disinvestment | - Stakeholder engagement - Educate patients and clinicians - Financial incentives for clinicians and other care providers |
| Hersh(59), 2004 | Interrupted time series | - None reported | - Evidence demonstrating that certain practices or treatments are harmful - Educating physicians and patients |
| Hicks(60), 2016 | Cohort of articles | - Lack of evidence - Lack of integration of evidence into clinical practice | - Educating physicians - Greater effort in monitoring and identifying low value practices - Communication between patient and care provider |
| Hillborne(61), 2014 | Guideline | - None reported | - Awareness of low-value practices - Stakeholder engagement - Communication between different professions (ex. Clinicians ordering tests and laboratory professionals) |
| Hines(62), 2015 | Case report/series | - Physicians’ perception that they are less responsible for lowering health care costs than others (ex. Insurance companies, lawyers, etc.) - Caregivers are not taught about healthcare costs during training - Slow-dissemination of evidence - Patient demand for services - Lack of incentives - Healthcare culture that emphasizes thoroughness over prudence - Level of training of caregivers | - Compensations (ex. Salary bonus) - Reduced concern for malpractice - Caregivers being able to tolerate or overcome uncertainty |
| Hislop(63), 2011 | Qualitative | - None reported | - Clinician led initiatives |
| Hobson(64), 2015 | Editorial/letter-to-the-editor | - Timeline issues (ex. If a project is large-scale, you need more time) - Vague goals (ex. What does it mean to change values and virtues?) - No reason or incentives to restrain doctor's activity - Tendency of institutions to implement quick fixes than taking the time to improve major procedures - Issues with payment model | - None reported |
| Hodgetts(65), 2012 | Mixed methods | - Lack of understanding of how to incorporate stakeholders into health policy decisions - Lack of consensus on what constitutes evidence | - Incorporating community and stakeholder perspectives |
| Hollingworth(66), 2015 | Mixed methods | - Lack of methods to identify low-value practices and technologies - Lack of evidence - Political, clinical, and social resistance - Lack of consensus as to what disinvestment means - Conflicting agendas between groups - Lack of resources - Perception that costs are the reason for restrictions - Difficulty creating a guideline because of variations in clinical practice - Not knowing how to include patients in the disinvestment process | - Monitoring treatments and services over time to examine how rates of usage have changed - Using benchmarking to identify low-value practices and to assess performance - Developing tools to help commissioners work with stakeholders |
| Hoverman(67), 2014 | Editorial/letter-to-the-editor | - Difficulty obtaining data for complex guidelines - Need for a multifaceted approach (ex. Engaging physicians, payers, etc.) - Lack of ways to measure the outcomes of certain practices | - Validating quality metrics - Encouraging collaboration amongst different groups - Generating feedback from stakeholders |
| Horvath(68), 2016 | Cohort of articles | - Doubt about the trustworthiness of Choosing Wisely recommendations - Lack of evidence | - None reported |
| Howard(69), 2012 | Predictive/economic modelling | - Willingness of physicians to abandon ineffective treatment | - None reported |
| Howard(70), 2011 | Interrupted time series | - Reluctance of specialists to give up on cutting-edge procedures - Lack of evidence | - Demand by patients and physicians to abandon low-value practices |
| Howard(71), 2015 | Editorial/letter-to-the-editor | - Absence of high quality evidence - Clinical trials are difficult to perform due to costs, time, and potential harm to patients - Sponsors do not want to sponsor an experiment that will harm their profits | - Using professional societies to make physicians consider costs and value of treatments - Changing the payment system to discourage low-value practices - Funding more and different types of research |
| Hsiao(72), 2009 | Cohort | - None reported | - Withdrawal of certain drugs from the market - Evidence-based reports |
| Huang(73), 2007 | Cohort | - None reported | - New evidence on treatments |
| Hughes(74), 2010 | Editorial/letter-to-the-editor | - Issues with patented vs generic drug brands - Costs of disinvestment and budget strains - People tend to not want to lose something they have, even if they get the chance to gain more in return | - New evidence on effectiveness, safety, or cost-effectiveness - Consulting stakeholders |
| Ibargoyen-Roteta(75), 2010 | Guideline | - Lack of dedicated resources to support and advance disinvestment policies - Lack of reliable methods to identify practices and technology based on clinical and cost effectiveness - Political, clinical, and social challenges - Lack of published studies proving ineffectiveness of current technologies and practices - Inadequate resources to support further research | - Gathering interested stakeholders - Incentives to promote disinvestment - Asking many decision makers to identify potential candidates of disinvestment |
| Ibargoyen-Roteta(76), 2009 | Other | - Lack of methods to identify and prioritize healthcare technologies | - Stakeholder engagement |
| Ioannidis(77), 2005 | Cohort of articles | - Conducting clinical research is time-consuming and challenging - May takes years to generate and publish data | - None reported |
| Ioannidis(78), 2013 | Editorial/letter-to-the-editor | - Financial and professional conflicts of interest - Missing evidence when comparing older and newer treatments | - None reported |
| Jackson(79), 2015 | Website, news item, professional society document | - Lack of political support, or support from decision makers - Disconnect between identifying low-value practices and implementing changes | - Clinician leadership and initiative |
| Johnson(80), 2015 | Editorial/letter-to-the-editor | - None reported | - Education of physicians outside a specific field (ex. Educating non-radiologists about low value practice in radiology) |
| Joshi(81), 2009 | Narrative review | - Difficulty controlling costs - De-implementation seen as threatening to practice and authority of physicians - Perception that what is new must be better - Lack of ways to identify problems and wasteful technology and practices - Ineffective technologies remain because there are no other options - Delay in implementing new evidence into practice | - Creating a new model for assessing health technology life cycle - Stakeholder engagement |
| Kahn(82), 2016 | Cohort | - Negative clinical trials do not change behaviours - Clinicians may not be influenced by guidelines or new evidence | - Publication of major guidelines - Financial incentives |
| Kelly(83), 2006 | Editorial/letter-to-the-editor | - De-investment may be difficult in areas where considerable investments have already been made - Disregard for scientific evidence - Lack of funding for public health research - Lack of methods to evaluate interventions | - None reported |
| Kiechle(84), 2014 | Narrative review | - None reported | - Formation of committees to evaluate value of laboratory tests using evidence based review of literature - Modifying requisition forms - Provide medical education on certain topics (ex. Grand rounds, newsletters, etc.) - Offering feedback and auditing clinician's use of new protocols |
| Koo(85), 2011 | Interrupted time series | - Culture and norms within an ICU - Specialty of the attending physician | - None reported |
| Kost(86), 2015 | Non-randomized controlled trial | - None reported | - Education for physicians - Creating a hospital culture committed to high-value practices |
| Krol(87), 2004 | Randomized controlled trial | - Lack of communication between prescribing physicians and patients | - Educating physicians - Financial incentives from insurance companies |
| Kulawik(88), 2009 | Before and after | - Lack of medical director's participation | - Access to education |
| Lasser(89), 2016 | Cohort | - Challenging to identify low-value practices | - None reported |
| Lawton(90), 2003 | Other | - None reported | - Reporting evidence for certain treatments |
| Leggett(91), 2012 | Mixed methods | - Lack of expertise in health technology reassessments - Political barriers - Lack of interests amongst stakeholders - Poor evidence base | - Government interest - Stakeholder engagement - Having a champion or strong leadership to provide support |
| Leggett(92), 2012 | Systematic evidence synthesis | - Resistance to change - Balancing interests and perspectives of multiple groups - Human and financial resources | - Stakeholder engagement - Knowledge transfer processes - Identifying and prioritizing health technologies and practices |
| Lesuis(93), 2017 | Before and after | - Previous knowledge and training - Since different clinicians have varying personalities (ex. Extraversion vs introversion), there needs to be a way to equally address everyone | - None reported |
| Lesuis(94), 2016 | Before and after | - None reported | - Educating physicians |
| Levin(95), 2011 | Other | - None reported | - Strong evidence - An ineffective practice can be substituted for another practice without negative effects - Creating a model to evaluate and manage technology implemention/de-implementation |
| Levinson(96), 2014 | Editorial/letter-to-the-editor | - Training patterns during medical school persists (ex. Students may be rewarded for ordering extra tests; medical schools rarely discourage ordering tests) - Desire to show thoroughness in a diagnosis | - Engaging patients in the conversation around de-implementation |
| MacFadden(97), 2015 | Editorial/letter-to-the-editor | - Lack of training in "resource stewardship" during medical school and residency | - Getting doctors to actively reflect upon the consequences of their clinical decisions |
| MacKean(98), 2013 | Consensus Panel | - Lack of leadership and top-down support - Lack of financial and human resources - Lack of expertise in health technology reassessment - Lack of development of tools to assess practices | - Stakeholder engagement - Creating health technology reassessment models that are adaptable to new contexts - Development of databases |
| Majumdar(99), 2004 | Interrupted time series | - None reported | - Published evidence that is credible - Declining patient demand - Declining promotions from companies |
| Malhotra(100), 2015 | Narrative review | - Work culture (ex. Doctors are pressured to focus less on open dialogue and more on meeting the demands of quality/outcome frameworks) - Payment systems (ex. Payment by results) - Lack of understanding of statistics amongst physicians - Lack of awareness of Choosing wisely - Specialist societies targeting simple procedures for recommendations and not targeting major procedures | - Development of tools that will help clinicians understand and share decisions based on evidence - Educating patients regarding risks and benefits - Greater involvement of patients in decision making |
| Makarov(101), 2015 | Cohort | - Regional, cultural, or local norms promoting certain practices | - None reported |
| Mason(102), 2015 | Editorial/letter-to-the-editor | - Patient demands and preferences - Lack of a rigorous process that identifies low-value care | - Educating patients |
| Massatti(103), 2008 | Mixed methods | - Maintaining support for policies even when leadership changes - Inadequate funding and staff training - Lack of stakeholder commitment - Lack of information technology integration - Lack of support from external agencies - Lack of knowledge on how to create and implement de-implementation policies | - Receiving guidance from external entities - Engagement in an in-depth planning process alongside community support |
| Mayer(104), 2015 | Systematic evidence synthesis | - Lack of human and financial resources - Lack of good evidence that identifies low-value practices - Lack of strong leadership from decision makers - Lack of a well-developed implementation strategy - Lack of patient involvement | - Identifying and assessing low-value practices - Stakeholder engagement - Tailoring strategies towards the interests of certain groups - Considering local contexts with regards to formulating recommendations - Communication and education |
| McCarthy(105), 2015 | Website, news item, professional society document | - Lack of interventions implemented - Awareness of guidelines is insufficient for change - Lack of evidence distinguishing low value and high value care | - None reported |
| McKinney(106), 2013 | Website, news item, professional society document | - Organizations are not collecting data about members' behaviours (ex. Are they implementing change?) - Fear of misdiagnosis - Lack of engagement amongst physicians and patients | - Disseminating information to patients |
| Mnatzaganian(107), 2015 | Predictive/economic modelling | - Lack of evidence - Perception that ordering a certain test is cheap - Concerns about missing an important diagnosis | - Greater role substitution (ex. Increasing the services of nurses) - Recommending a restricted set of tests |
| Montini(108), 2015 | Narrative review | - Financial structure (ex. Stakeholders lose revenue if a service they provide is de-implemented) - Hard to de-implement if someone (ex. Government or insurance) continues to reimburse for that service or treatment - Competition and tensions between different specialties | - Development of alternate treatments to replace the treatment being de-implemented - Patient awareness of treatment option - Using social sciences (ex. Psychology) to design ways to implement change - Changing the social, political, and economic aspects of healthcare rather than individuals |
| Morden(109), 2014 | Editorial/letter-to-the-editor | - Lack of methods to measure quality outcomes - Need to find a balance between financial incentives and de-implementing low-value practices | - Linking de-implementation to financial incentives - Audits and improvement tasks - Public education and reporting - Improved communication between physician and patient, thus deterring litigation and patient dissatisfaction |
| Mortimer(110), 2010 | Narrative review | - Difficulty identifying treatments that can be de-invested - Financial and budgetary pressure - Taking the path of least resistance when trying to implement disinvestment plans - Failure to develop a comprehensive disinvestment plan - Personal interests of stakeholders (ex. Oncologists don't want to give up part of their budget to help other departments) - Lack of incentives and benefits - Lack of equal representation on advisory councils | - Genuine interest of decision makers |
| Moscucci(111), 2011 | Editorial/letter-to-the-editor | - Lack of awareness, familiarity, and agreement on the evidence supporting guidelines - Harder to eliminate a behaviour than to implement a new one - Patient preferences - Environmental-barriers | - None reported |
| Moynihan(112), 2012 | Editorial/letter-to-the-editor | - Difficulty finding evidence (ex. Lack of published literature) | - None reported |
| Murphy(113), 2013 | Cohort | - Lack of provider awareness and agreement with evidence - Organization policies - Staffing and interpersonal communication problems | - None reported |
| Murphy(114), 2015 | Editorial/letter-to-the-editor | - Disseminating information to a large group of people - Topics chosen for de-implementation need support from stakeholders - Topics are chosen based on rigorous criteria - Lack of public awareness | - Using benchmarking and feedback to monitor and implement change |
| Murphy(115), 2015 | Editorial/letter-to-the-editor | - None reported | - Being familiar with Choosing Wisely guidelines for non-physicians (ex. Nurses, midwives, etc.) |
| Mutter(116), 2016 | Editorial/letter-to-the-editor | - Time capacity - Lack of patients who are interested in participating in studies to assess effectiveness of treatment | - None reported |
| Naik(117), 2013 | Qualitative | - Medical-legal considerations - Lack of incentives | - None reported |
| Naylor(118), 2004 | Editorial/letter-to-the-editor | - Resources go towards advertisement of drugs, rather than on scientific research on the effects of drugs - Less attention is given to drugs that have recently moved off patent | - None reported |
| Neeman(119), 2012 | Interrupted time series | - Cost consciousness may not be taught during caregiver’s education | - Providing feedback to physicians |
| Niven(120), 2016 | Narrative review | - Perceived threat on one's autonomy - Lack of rigorous evidence and testing to show whether a treatment is effective or not - Lack of an objective method to identify and evaluate low-value practices - Reluctance to part ways with established methods of providing care - Variation amongst physicians (ex. Older physicians may be less willing to de-implement than younger physicians) | - Changes to policy - Changes to funding model - Early stakeholder engagement - Evaluating the effectiveness of interventions |
| Noseworthy(121), 2012 | Editorial/letter-to-the-editor | - Definition of 'disinvestment' may be seen as a polarizing and pejorative term | - Using incentives |
| Pandey(122), 2016 | Cohort | - Recommending guidelines is not enough to elicit change in practices | - None reported |
| Parkinson(123), 2015 | Systematic evidence synthesis | - The payment system - Lack of clear criteria to identify low-value practices - Perverse incentives (ex. Physicians get paid if they use a certain drug) - Perception that change will render previous medical training obsolete - Lack of alternate treatments should a low-value practice be de-implemented | - Identifying low value practices - Financial incentives - Communicating with stakeholders about the de-implementation process, what evidence is required, pros vs cons, alternative methods, etc. |
| Parks(124), 2016 | Narrative review | - Lack of communication skills amongst clinicians - Lack of number sense (ex. Overestimating or underestimating benefits/drawbacks) | - None reported |
| Pearson(125), 2007 | Narrative review | - Stakeholders are enthusiastic about dis-investment, but when asked for suggestions, they are relatively silent | - Developing criteria to prioritize and identify ineffective treatments and technology - Reliable evidence - Cooperation amongst different agencies to develop guidelines to aid in disinvestment - Framing disinvestment guidelines as recommendations. - Creating a central site for data storage and collection |
| Pisetsky(126), 2013 | Editorial/letter-to-the-editor | - The items on Choosing wisely may not be radical or controversial - Confusing symptoms (ex. Back pain may have different symptoms present, so it is difficult to implement guidelines when the physicians don't know what to look for) - Tendency for Choosing wisely recommendations to be "don’ts" | - Greater communication between doctor and patient regarding treatments |
| Polisena(127), 2013 | Systematic evidence synthesis | - Lack of incentives - Health professionals may view de-investment differently (ex. Reduction of health care services) - Difficulty ensuring that requests from all departments are considered - Which allocation decisions are made | - Training healthcare professionals on economical and ethical principles - Review of investment and disinvestment opportunities - Utilizing weights when developing decision criteria to reduce the subjectivity of the recommendations |
| Prasad(128), 2014 | Narrative review | - Evidence wars (ex. Benefits vs drawbacks) | - Systematically testing treatments with little to no evidence - Prioritize and identify which practices are most harmful, which has alternatives, and which will lessen the financial burden - Having strong and robust data to support decisions |
| Raghavan(129), 2015 | Website, news item, professional society document | - Framing de-implementation as an issue of cost-containment - Misunderstanding what statistical significance and clinical relevance means | - Reporting data transparently |
| Raman(130), 2017 | Editorial/letter-to-the-editor | - Unclear guidelines despite strong evidence | - Institutional policies and audits |
| Ramsey(131), 2015 | Case control | - None reported | - Studying the differences in overall adherence and cost before decision making or policy development |
| Rao(132), 2012 | Editorial/letter-to-the-editor | - Malpractice liability - Fear of misdiagnosis - Patient demands - Physicians want to generate revenue - No communication between healthcare teams (ex. A patient transfers to a new doctor; the new doctor orders the same tests as the old doctor did) | - Educating other health professionals (ex. Radiologists educating non-radiologists about which tests to order) - Computerized decision-support tools - Increasing awareness coupled with greater physician knowledge |
| Rao(133), 2014 | Narrative review | - None reported | - Increasing awareness through educational sessions, involvement of Consumer Report, etc. |
| Read(134), 2016 | Editorial/letter-to-the-editor | - Not knowing the reasons for why physicians don't follow guidelines | - None reported |
| Resnick(135), 2013 | Editorial/letter-to-the-editor | - None reported | - Involving more health care professionals (ex. Nurses, because they are by the bedside a lot and engage in many conversations with patients and family) |
| Robert(136), 2014 | Qualitative | - Legal and licensing issues for newer, less expensive drugs - Lack of understanding of what the barriers and facilitators are towards de-implementation - Lack of evidence that identifies low-value practices - Pursuing de-implementation solely for the purpose of cutting costs - Not implementing an evidence-based approach towards de-implementation   Difficulty collecting good data | - Clinical backing and strong leadership - Stakeholder engagement - Strong leadership and political support - Clinical and patient support - Clear rationale for strategies and change - Engage stakeholder early |
| Rohren(137), 2014 | Editorial/letter-to-the-editor | - Lack of evidence showing how effective a practice is - Lack of consensus as to what high-level evidence looks like | - Stakeholder engagement and collaboration |
| Roman(138), 2014 | Narrative review | - Financial interest of stakeholders - Optimism bias (ie. Willingness to accept new information in their favour than out of it) - Loss aversion (ie. Feeling more strongly about losses than gains) | - Improved evaluation and monitoring of drugs - Greater communication between doctor and patient |
| Rooshenas(139), 2015 | Qualitative | - Lack of confidence in decision making - Lack of strong leadership and political support - Lack of consensus as to what disinvestment means - Financial and budget concerns (ex. lack of funding) - Lack of information (ex. evidence on low-value practices) and guidance - Time capacity - Work environment (ex. some hospitals hinder collaboration) - Resistance from certain groups (ex. hospital managers) - The way "disinvestment" was framed | - Clinical collaboration amongst different groups - Stakeholder engagement |
| Rosenberg(140), 2015 | Cohort | - Lack of public involvement when implementing evidence - Lack of tools to gauge how well evidence is being implemented | - Physician communication training - Data feedback - Patient-focussed strategies - Financial incentives - Systematic interventions (ex. Clinical decision support in electronic medical records) |
| Schwartz(141), 2015 | Interrupted time series | - Regional difference or cultural differences amongst different places | - Alternate payment models |
| Scott(142), 2014 | Cohort | - None reported | - Improved awareness of wasteful practices - Electronic decision support |
| Sharp(143), 2015 | Cohort | - None reported | - Greater understanding of how to coordinate services and how different settings implement guidelines - Physician education - Implementing clinical decision aids into certain places (ex. Electronic health record) |
| Silverstein(144), 2016 | Cross-sectional | - None reported | - Good communication between physician and patient - Educational material for patients |
| Simos(145), 2015 | Cohort | - Dissemination of clinical guidelines alone is an ineffective way to change physician behaviours - Lack of knowledge translation strategies | - None reported |
| Siontis(146), 2009 | Cohort of articles | - Defending one's own interests (ex. A specialist defending his/her interests because of their relationship with certain companies) | - None reported |
| Siwek(147), 2014 | Editorial/letter-to-the-editor | - Difficulty incorporating evidence into practice - Enhancing evidence base for recommendations | - Using a multifaceted approach (ex. Social media, scientific journals, etc.) - Creating a unique and easily accessible database |
| Sorrel(148), 2012 | Website, news item, professional society document | - Patient expectations - Physicians' habits - Too much evidence for doctors to track and understand - Concern that insurance companies may view guidelines as a standard to refuse certain services | - Making information (ex. Evidence about treatments) more accessible |
| Speight(149), 2015 | Website, news item, professional society document | - Misunderstanding Choosing Wisely recommendations | - None reported |
| Stafford(150), 2004 | Interrupted time series | - None reported | - Patients visiting physicians to discuss treatments - Decrease in drug promotion - Recommendations receive more rapid responses when trials reveal negative rather than positive results |
| Street(151), 2011 | Qualitative | - Lack of understanding of socio-political contexts - Patient dissatisfaction with cost of services | - Community and stakeholder involvement |
| Sukel(152), 2008 | Before and after | - None reported | - Communication between physicians and patients |
| Surial(153), 2015 | Cohort | - Lack of high quality evidence | - Awareness of benefits and drawbacks of treatments |
| Tanaka(154), 2016 | Narrative review | - Multiple guidelines made by different institutions, with no coordination between them - Patient preference and demand - Lack of time in communicating with patients - Patients unaware of benefits and risks with certain procedures | - Education interventions - Computer-based feedback |
| Tatsioni(155), 2007 | Cohort of articles | - Delay in accepting new evidence showing an intervention is ineffective | - None reported |
| Tatsioni(156), 2010 | Cohort of articles | - Medical literature continues to support previous practices - Industry ties of professional societies | - None reported |
| Thiebaud(157), 2006 | Cohort | - None reported | - New evidence on treatments |
| Urness(158), 2016 | Website, news item, professional society document | - Cognitive dissonance between medical training and emerging evidence - Physician's lack of knowledge - Relationship influences - Fear of harming patients - Lawsuits | - Availability of evidence and guidelines - Education, audits and feedback - Considering the well-being of the patient |
| Vogel(159), 2016 | Website, news item, professional society document | - None reported | - Need to pair education with structural change - Integrating recommendations into computer systems, screensavers, blogs, etc. - Enforcement of a policy |
| Vogel(160), 2015 | Narrative review | - Concerns about how patients will perceive de-implementation initiatives | - Programming recommendations into the electronic health record - Educational interventions |
| Vogel(161), 2015 | Website, news item, professional society document | - Fear of litigation - Patient expectations - Habits of caregivers | - Implementing a decision support tool - Incorporating Choosing wisely into medical school curriculums |
| Volpp(162), 2012 | Editorial/letter-to-the-editor | - Physicians and patients may not understand what procedures are low value | - Changing how consumers pay for their insurance |
| Voorn(163), 2012 | Mixed methods | - Lack of detailed understanding of how barriers and facilitators influence medical professionals' behaviours | - Educational outreach or interactive educational strategies |
| Voorn(164), 2014 | Randomized controlled trial | - Lack of alternatives - Lack of interest in gaining more information on low-value practices - Lack of motivation in saving money or benefitting others - Concern for patient safety - Pressure from suppliers - Lack of influence from individual physicians | - Interactive education for clinicians - Feedback on clinician's practice - Spreading information to societies, thus making evidence more available |
| Wang(165), 2015 | Other | - Commercial entities resist evidence because it harms their profits - Academics and specialist are resistant towards new evidence - Lack of initiative and support from specialist societies | - Greater involvement of method experts and non-specialists when evaluating evidence |
| Watt(166), 2012 | Mixed methods | - Multiple perspectives to consider | - Stakeholder engagement |
| Watt(167), 2012 | Other | - Lack of consensus on what the problem is - Communication problems between experts and policy makers | - Stakeholder engagement |
| Wolfson(168), 2016 | Narrative review | - Using an inappropriate strategy (ex. Broad engagement of societies instead of controlling them) | - Respecting the autonomy of practitioners - Educating doctors on how to communicate with patients regarding overuse - If one society supports an initiative, others may follow along |
| Wolfson(169), 2016 | Editorial/letter-to-the-editor | - Physician's perception that they are not responsible for reducing costs - Lack of resources and time - Lack of communication skills when discussing benefits and costs | - None reported |
| Wolfson(170), 2014 | Narrative review | - Patient demands and preferences - Failure to garner media attention - Resistance from stakeholders due to personal interests - Many physicians require guidelines on how to communicate with patients | - Greater communication between doctor and patient regarding treatments - Carefully framing issues so that it appeals to physicians and patients - Having strong leadership support from physicians and specialty societies - Stakeholder engagement |
| Wong(171), 2014 | Narrative Review | - Physicians feel stressed about communicating with patients regarding benefits and risks of treatments - No consensus as to whether or not certain recommendations should be de-implemented | - New ways to offer services (ex. Do it yourself tests) |
| Zikmund-Fisher(172), 2017 | Cross-sectional | - Fear of medical malpractice - Patient requests for tests and treatments - Lack of shared decision making time with patients - Lack of support and resources to determine if a patient would benefit from a treatment or service - System that rewards quantity over quality - Fear of patient resistance to Choosing wisely recommendations | - Communicating with patients |

**References**

1. Admon AJ, Cooke CR. Will Choosing Wisely® Improve Quality and Lower Costs of Care for Patients with Critical Illness? Annals of the American Thoracic Society. 2014;11(5):823-7.

2. Agiro A, DeVries A, Rosenberg A. Choosing Wisely Recommendations Using Administrative Claims Data—Reply. JAMA Internal Medicine. 2016;176(4):566-.

3. Angus DC. Ongoing Use of Pulmonary Artery Catheters Despite Negative Trial Findings. JAMA Internal Medicine. 2016;176(1):133-.

4. Archbald-Pannone LR, Nadkarni M. Elderly Adults Not Chosen for Society for General Internal Medicine Choosing Wisely Recommendation. 2016. p. 693-4.

5. Arseneau JP. Choosing Wisely Canada recommendations: Interview with Dr Jean Pierre Arseneau. Canadian family physician Medecin de famille canadien. 2016;62(5):408-.

6. Atwater BD, Oujiri J, Wolff MR. The immediate impact of the Clinical Outcomes Utilizing Revascularization and Aggressive Drug Evaluation (COURAGE) trial on the management of stable angina. Clin Cardiol. 2009;32(8):E1-3.

7. Azermai M, Vander Stichele RRH, Van Bortel LM, Elseviers MM. Barriers to antipsychotic discontinuation in nursing homes: an exploratory study. Aging & Mental Health. 2014;18(3):346-53.

8. Balekian AA, Fisher JM, Gould MK. Brain Imaging for Staging of Patients With Clinical Stage IA Non-small Cell Lung Cancer in the National Lung Screening Trial. Chest. 2016;149(4):943-50.

9. Banta HD. The Case for Reassessment of Health Care Technology. JAMA. 1990;264(2):235.

10. Bauman G. Choosing Wisely? “It’s Complicated!”. Practical Radiation Oncology. 2016;6(2):71-3.

11. Beller GA. Tests that may be overused or misused in cardiology: The Choosing Wisely ® campaign. Journal of Nuclear Cardiology. 2012;19(3):401-3.

12. Bernstein J. Not the Last Word: Choosing Wisely. Clinical Orthopaedics and Related Research®. 2015;473(10):3091-7.

13. Bhatia RS, Levinson W, Shortt S, Pendrith C, Fric-Shamji E, Kallewaard M, et al. Measuring the effect of Choosing Wisely: an integrated framework to assess campaign impact on low-value care. BMJ Quality & Safety. 2015;24(8):523-31.

14. Blumenthal-Barby JS. "Choosing Wisely" to Reduce Low-Value Care: A Conceptual and Ethical Analysis. Journal of Medicine and Philosophy. 2013;38(5):559-80.

15. Borasio GD, Jox RJ. Choosing wisely at the end of life: the crucial role of medical indication. Swiss Medical Weekly. 2016;146(November):w14369-w.

16. Brunt ME, Murray MD, Hui SL, Kesterson J, Perkins AJ, Tierney WM. Mass media release of medical research results: an analysis of antihypertensive drug prescribing in the aftermath of the calcium channel blocker scare of March 1995. J Gen Intern Med. 2003;18(2):84-94.

17. Callaghan BC, De Lott LB, Kerber KA, Burke JF, Skolarus LE. Neurology Choosing Wisely recommendations. Neurology: Clinical Practice. 2015;5(5):439-47.

18. Casarett D. The Science of Choosing Wisely — Overcoming the Therapeutic Illusion. New England Journal of Medicine. 2016;374(13):1203-5.

19. Clement F, Charlton B. Challenges in Choosing Wisely’s International Future. JAMA Internal Medicine. 2015;175(4):644-.

20. Colla CH, Kinsella EA, Morden NE, Meyers DJ, Rosenthal MB, Sequist TD. Physician perceptions of Choosing Wisely and drivers of overuse. The American journal of managed care. 2016;22(5):337-43.

21. Colla CH, Morden NE, Sequist TD, Schpero WL, Rosenthal MB. Choosing Wisely: Prevalence and Correlates of Low-Value Health Care Services in the United States. Journal of General Internal Medicine. 2015;30(2):221-8.

22. Colla CH, Sequist TD, Rosenthal MB, Schpero WL, Gottlieb DJ, Morden NE. Use of non-indicated cardiac testing in low-risk patients: Choosing Wisely. BMJ Quality & Safety. 2015;24(2):149-53.

23. Cooper C, Starkey K. Disinvestment in health care. BMJ. 2010;340:c1413.

24. Crosby J. Choosing Wisely Canada recommendations. Canadian family physician Medecin de famille canadien. 2016;62(7):568-.

25. Davidoff F. On the Undiffusion of Established Practices. JAMA Internal Medicine. 2015;175(5):809-.

26. de Boer MJ, van der Wall EE. Choosing wisely or beyond the guidelines. Netherlands Heart Journal. 2013;21(1):1-2.

27. Deyell MW, Buller CE, Miller LH, Wang TY, Dai D, Lamas GA, et al. Impact of National Clinical Guideline recommendations for revascularization of persistently occluded infarct-related arteries on clinical practice in the United States. Arch Intern Med. 2011;171(18):1636-43.

28. Donaldson C, Bate A, Mitton C, Dionne F, Ruta D. Rational disinvestment. QJM. 2010;103(10):801-7.

29. Dorsey ER. Choosing Wisely: You get what you pay for. Neurology. 2013;81(11):946-7.

30. Edwards RT, Charles JM, Thomas S, Bishop J, Cohen D, Groves S, et al. A national Programme Budgeting and Marginal Analysis (PBMA) of health improvement spending across Wales: disinvestment and reinvestment across the life course. BMC Public Health. 2014;14(1):837-.

31. Elshaug AG, Hiller JE, Moss JR. Exploring policy-makers' perspectives on disinvestment from ineffective healthcare practices. 2008. p. 1-9.

32. Elshaug AG, Hiller JE, Tunis SR, Moss JR. Challenges in Australian policy processes for disinvestment from existing, ineffective health care practices. Australia and New Zealand Health Policy. 2007;4(1):23-.

33. Elshaug AG, Watt AM, Moss JR, Hiller JE. Policy Perspectives on the Obsolescence of Health Technologies in Canada. 2009.

34. Elshaug AG, Moss JR, Littlejohns P, Karnon J, Merlin TL, Hiller JE. Identifying existing health care services that do not provide value for money. The Medical journal of Australia. 2009;190(5):269-73.

35. Elshaug AG, McWilliams JM, Landon BE. The value of low-value lists. 2013. p. 775-6.

36. Ferguson T. Improving Health Outcomes and Promoting Stewardship of Resources: ABIM Foundation’s Choosing Wisely Campaign. American Medical Association Journal of Ethics. 2012;14(11):880-4.

37. Ferrari R. Evaluation of the Canadian Rheumatology Association Choosing Wisely recommendation concerning anti-nuclear antibody (ANA) testing. Clinical Rheumatology. 2015;34(9):1551-6.

38. Ferrari R, Prosser C. Testing Vitamin D Levels and Choosing Wisely. JAMA Internal Medicine. 2016;176(7):1019-.

39. Fessele K. Are You Choosing Wisely in Your Professional Practice? Clinical Journal of Oncology Nursing. 2016;20(2):211-2.

40. Fleisher LA. Preoperative Consultation Before Cataract Surgery. JAMA Internal Medicine. 2014;174(3):389-.

41. Fritzler MJ. Choosing wisely: Review and commentary on anti-nuclear antibody (ANA) testing. Autoimmunity Reviews. 2016;15(3):272-80.

42. Garner S, editor Disinvestment: thre UK experience. International Socierty for Pharmacoeconomics and Outcomes Research, 15th Annual European Congress; 2012.

43. Garner S, Littlejohns P. Disinvestment from low value clinical interventions: NICEly done? BMJ (Online). 2011;343(7819):d4519-d.

44. Gerdvilaite J, Nachtnebel A, editors. Disinvestment. Overview of disinvestment experiences and challenges in selected countries2011.

45. Gershengorn HB, Wunsch H. Understanding Changes in Established Practice. Critical Care Medicine. 2013;41(12):2667-76.

46. Gidwani R, Sinnott P, Avoundjian T, Lo J, Asch SM, Barnett PG. Inappropriate ordering of lumbar spine magnetic resonance imaging: are providers Choosing Wisely? The American journal of managed care. 2016;22(2):e68-76.

47. Glauser W. Choosing Wisely campaign well received. Canadian Medical Association Journal. 2014;186(8):E239-E40.

48. Gnjidic D, Elshaug AG. De-adoption and its 43 related terms: harmonizing low-value care terminology. BMC Medicine. 2015;13(1):273-.

49. Greene SE, Massone R. A survey of emergency medicine residents’ perspectives of the choosing wisely campaign. The American Journal of Emergency Medicine. 2015;33(6):853-5.

50. Haas JS, Kaplan CP, Gerstenberger EP, Kerlikowske K. Changes in the Use of Postmenopausal Hormone Therapy after the Publication of Clinical Trial Results. Annals of Internal Medicine. 2004;140(3):184-.

51. Haas M, Hall J, Viney R, Gallego G. Breaking up is hard to do: why disinvestment in medical technology is harder than investment. Aust Health Rev. 2012;36(2):148-52.

52. Haines T, O'Brien L, McDermott F, Markham D, Mitchell D, Watterson D, et al. A novel research design can aid disinvestment from existing health technologies with uncertain effectiveness, cost-effectiveness, and/or safety. Journal of Clinical Epidemiology. 2014;67(2):144-51.

53. Halpern SD, Becker D, Curtis JR, Fowler R, Hyzy R, Kaplan LJ, et al. An Official American Thoracic Society/American Association of Critical-Care Nurses/American College of Chest Physicians/Society of Critical Care Medicine Policy Statement: The Choosing Wisely® Top 5 List in Critical Care Medicine. American Journal of Respiratory and Critical Care Medicine. 2014;190(7):818-26.

54. Harris E ML, Hewson K, Jacobsen N. Disinvestment in Australia and New Zealand.: HealthPACT; 2013.

55. Harvey E. Choosing Wisely (and carefully) Canada. Canadian Journal of Surgery. 2014;57(3):149-.

56. Hauptman PJ, Schnitzler MA, Swindle J, Burroughs TE. Use of Nesiritide Before and After Publications Suggesting Drug-Related Risks in Patients With Acute Decompensated Heart Failure. JAMA. 2006;296(15):1877-.

57. Hawasli AH, Chicoine MR, Dacey RG. Choosing Wisely. Neurosurgery. 2015;76(1):1-6.

58. Henshall C, Schuller T, Mardhani-Bayne L. Using health technology assessment to support optimal use of technologies in current practice: the challenge of “disinvestment”. International Journal of Technology Assessment in Health Care. 2012;28(3):203-10.

59. Hersh AL, Stefanick ML, Stafford RS. National use of postmenopausal hormone therapy. ACC Current Journal Review. 2004;13(4):18-.

60. Hicks LK, Rajasekhar A, Bering H, Carson KR, Kleinerman J, Kukreti V, et al. Identifying existing Choosing Wisely recommendations of high relevance and importance to hematology. American Journal of Hematology. 2016;91(8):787-92.

61. Hillborne L. Choosing Wisely: selecting the right

test for the right patient at the right

time. 2014.

62. Hines JZ, Sewell JL, Sehgal NL, Moriates C, Horton CK, Chen AH. “Choosing Wisely” in an Academic Department of Medicine. American Journal of Medical Quality. 2015;30(6):566-70.

63. Hislop JM. PHP129 Societal Preferences for Health Technology Disinvestment Policy: Views of Scottish Taxpayers &#x2013; A Qualitative Study. Value in Health. 2011;14(7):A356-A7.

64. Hobson C. Choosing Wisely? Not as the Academy of Medical Royal Colleges envisages. British Journal of Hospital Medicine. 2015;76(12):676-7.

65. Hodgetts K, Elshaug AG, Hiller JE. What counts and how to count it: Physicians’ constructions of evidence in a disinvestment context. Social Science & Medicine. 2012;75(12):2191-9.

66. Hollingworth W, Rooshenas L, Busby J, Hine CE, Badrinath P, Whiting PF, et al. Using clinical practice variations as a method for commissioners and clinicians to identify and prioritise opportunities for disinvestment in health care: a cross-sectional study, systematic reviews and qualitative study. Health Services and Delivery Research. 2015;3(13):1-172.

67. Hoverman JR. Getting From Choosing Wisely to Spending Wisely. Journal of Oncology Practice. 2014;10(3):223-5.

68. Horvath K, Semlitsch T, Jeitler K, Abuzahra ME, Posch N, Domke A, et al. Choosing Wisely: assessment of current US top five list recommendations’ trustworthiness using a pragmatic approach. BMJ Open. 2016;6(10):e012366-e.

69. Howard DH, Shen Y-C. Comparative Effectiveness Research, Technological Abandonment, and Health Care Spending. In: Bolin K, Kaestner R, editors. The Economics of Medical Technology. Advances in Health Economics and Health Services Research. 23: Emerald Group Publishing Limited; 2012. p. 103-21.

70. Howard DH, Kenline C, Lazarus HM, LeMaistre CF, Maziarz RT, McCarthy Jr PL, et al. Abandonment of High-Dose Chemotherapy/Hematopoietic Cell Transplants for Breast Cancer Following Negative Trial Results. Health Services Research. 2011;46(6pt1):1762-77.

71. Howard DH, Gross CP. Producing Evidence to Reduce Low-Value Care. JAMA internal medicine. 2015;175(12):1893-4.

72. Hsiao F-Y, Tsai Y-W, Huang W-F. Changes in physicians' practice of prescribing cyclooxygenase-2 inhibitor after market withdrawal of rofecoxib: A retrospective study of physician-patient pairs in Taiwan. Clinical Therapeutics. 2009;31(11):2618-27.

73. Huang W-F, Tsai Y-W, Hsiao F-Y, Liu W-C. Changes of the prescription of hormone therapy in menopausal women: An observational study in Taiwan. BMC Public Health. 2007;7(1):56-.

74. Hughes DA, Ferner RE. New drugs for old: disinvestment and NICE. BMJ. 2010;340(feb25 1):c572-c.

75. Ibargoyen-Roteta N, Gutiérrez-Ibarluzea I, Asua J. Guiding the process of health technology disinvestment. Health Policy. 2010;98(2-3):218-26.

76. Ibargoyen-Roteta N, Gutierrez-Ibarluzea I, Asua J, Benguria-Arrate G, Galnares-Cordero L. Scanning the horizon of obsolete technologies: Possible sources for their identification. International Journal of Technology Assessment in Health Care. 2009;25(03):249-54.

77. Ioannidis JPA. Contradicted and Initially Stronger Effects in Highly Cited Clinical Research. JAMA. 2005;294(2):218-.

78. Ioannidis JPA. In reply II—Reversal of Medical Practices. Mayo Clinic Proceedings. 2013;88(10):1184-.

79. Jackson C. Shaping our own destiny by Choosing Wisely. Australian family physician. 2015;44(6):425-6.

80. Johnson PT, Mahesh M, Fishman EK. Image Wisely and Choosing Wisely: Importance of Adult Body CT Protocol Design for Patient Safety, Exam Quality, and Diagnostic Efficacy. Journal of the American College of Radiology. 2015;12(11):1185-90.

81. Joshi N, Stahnisch F, Noseworthy T. Reassessment of Health Technologies: Obsolescence and Waste. 2009. Report No.: 9781926680286.

82. Kahn JM, Le TQ. Adoption and de-adoption of drotrecogin alfa for severe sepsis in the United States. Journal of Critical Care. 2016;32:114-9.

83. Kelly M. Public health programmes and interventions and disinvestmentr: National Institute for Health and Care Excellence; 2006 [

84. Kiechle FL, Arcenas RC, Rogers LC. Establishing benchmarks and metrics for disruptive technologies, inappropriate and obsolete tests in the clinical laboratory. Clinica Chimica Acta. 2014;427:131-6.

85. Koo KKY, Sun JCJ, Zhou Q, Guyatt G, Cook DJ, Walter SD, et al. Pulmonary artery catheters: Evolving rates and reasons for use*. Critical Care Medicine. 2011;39(7):1613-8.

86. Kost A, Genao I, Lee JW, Smith SR. Clinical Decisions Made in Primary Care Clinics Before and After Choosing Wisely. The Journal of the American Board of Family Medicine. 2015;28(4):471-4.

87. Krol N, Wensing M, Haaijer-Ruskamp F, Muris JWM, Numans ME, Schattenberg G, et al. Patient-directed strategy to reduce prescribing for patients with dyspepsia in general practice: a randomized trial. Alimentary Pharmacology and Therapeutics. 2004;19(8):917-22.

88. Kulawik D, Sands JJ, Mayo K, Fenderson M, Hutchinson J, Woodward C, et al. Focused Vascular Access Education to Reduce the Use of Chronic Tunneled Hemodialysis Catheters: Results of a Network Quality Improvement Initiative. Seminars in Dialysis. 2009;22(6):692-7.

89. Lasser EC, Pfoh ER, Chang HY, Chan KS, Bailey JC, Kharrazi H, et al. Has Choosing Wisely® affected rates of dual-energy X-ray absorptiometry use? Osteoporosis International. 2016;27(7):2311-6.

90. Lawton B. Changes in use of hormone replacement therapy after the report from the Women's Health Initiative: cross sectional survey of users. BMJ. 2003;327(7419):845-6.

91. Leggett LE, Mackean G, Noseworthy TW, Sutherland L, Clement F. Current status of health technology reassessment of non-drug technologies: survey and key informant interviews. Health Research Policy and Systems. 2012;10(1):38-.

92. Leggett L, Noseworthy TW, Zarrabi M, Lorenzetti D, Sutherland LR, Clement FM. Health technology reassessment of non-drug technologies: current practices. Int J Technol Assess Health Care. 2012;28(3):220-7.

93. Lesuis N, den Broeder AA, van Vollenhoven RF, Vriezekolk JE, Hulscher M. Choosing wisely in daily practice: a mixed methods study on determinants of antinuclear antibody testing by rheumatologists. Scandinavian Journal of Rheumatology. 2017;46(3):241-6.

94. Lesuis N, Hulscher MEJL, Piek E, Demirel H, van der Laan-Baalbergen N, Meek I, et al. Choosing Wisely in Daily Practice: An Intervention Study on Antinuclear Antibody Testing by Rheumatologists. Arthritis Care & Research. 2016;68(4):562-9.

95. Levin L. Disinvestment Strategies Based on Evidence Guided Adoption and Obsolescence of Technologies: The Ontario Experience. Montreal; 2011.

96. Levinson W, Huynh T. Engaging physicians and patients in conversations about unnecessary tests and procedures: Choosing Wisely Canada. Canadian Medical Association Journal. 2014;186(5):325-6.

97. MacFadden DR, Gold WL, Al-Busaidi I, Craig JD, Petrescu D, Saltzman IS, et al. An Educational Forum to Engage Infectious Diseases and Microbiology Residents in Resource Stewardship Modelled after the Choosing Wisely Campaign. Canadian Journal of Infectious Diseases and Medical Microbiology. 2015;26(5):231-3.

98. MacKean G, Noseworthy T, Elshaug AG, Leggett L, Littlejohns P, Berezanski J, et al. Health technology reassessment: the art of the possible. International Journal of Technology Assessment in Health Care. 2013;29(4):418-23.

99. Majumdar SR. Promotion and Prescribing of Hormone Therapy After Report of Harm by the Women’s Health Initiative. JAMA. 2004;292(16):1983-.

100. Malhotra A, Maughan D, Ansell J, Lehman R, Henderson A, Gray M, et al. Choosing Wisely in the UK: the Academy of Medical Royal Colleges' initiative to reduce the harms of too much medicine. BMJ. 2015;350(may12 7):h2308-h.

101. Makarov DV, Soulos PR, Gold HT, Yu JB, Sen S, Ross JS, et al. Regional-Level Correlations in Inappropriate Imaging Rates for Prostate and Breast Cancers. JAMA Oncology. 2015;1(2):185-.

102. Mason DJ. Choosing wisely: Changing clinicians, patients, or policies? 2015. p. 657-8.

103. Massatti RR, Sweeney HA, Panzano PC, Roth D. The De-adoption of Innovative Mental Health Practices (IMHP): Why Organizations Choose not to Sustain an IMHP. Administration and Policy in Mental Health and Mental Health Services Research. 2008;35(1-2):50-65.

104. Mayer J, Nachtnebel A. Disinvesting from ineffective technologies: lessons learned from current programs. International Journal of Technology Assessment in Health Care. 2015;31(6):355-62.

105. McCarthy M. US Choosing Wisely campaign has had only modest success, study finds. BMJ. 2015;351(October):h5437-h.

106. McKinney M. Curbing overuse. Providers hail value of Choosing Wisely, but skeptics say initiative comes with risk. Modern healthcare. 2013;43(8):1-5.

107. Mnatzaganian G, Karnon J, Moss JR, Elshaug AG, Metz M, Frank OR, et al. Informing disinvestment with limited evidence: cobalamin deficiency in the fatigued. International Journal of Technology Assessment in Health Care. 2015;31(3):188-96.

108. Montini T, Graham ID. “Entrenched practices and other biases”: unpacking the historical, economic, professional, and social resistance to de-implementation. Implementation Science. 2015;10(1):24-.

109. Morden NE, Colla CH, Sequist TD, Rosenthal MB. Choosing Wisely — The Politics and Economics of Labeling Low-Value Services. New England Journal of Medicine. 2014;370(7):589-92.

110. Mortimer D. Reorienting programme budgeting and marginal analysis (PBMA) towards disinvestment. BMC Health Services Research. 2010;10(1):288-.

111. Moscucci M. Medical Reversal, Clinical Trials, and the “Late” Open Artery Hypothesis in Acute Myocardial Infarction. Archives of Internal Medicine. 2011;171(18):1643-.

112. Moynihan RN. A healthy dose of disinvestment. Medical Journal of Australia. 2012;196(3):158-.

113. Murphy DJ, Needham DM, Netzer G, Zeger SL, Colantuoni E, Ness P, et al. RBC Transfusion Practices Among Critically Ill Patients. Critical Care Medicine. 2013;41(10):2344-53.

114. Murphy MF. The Choosing Wisely campaign to reduce harmful medical overuse: its close association with Patient Blood Management initiatives. Transfusion Medicine. 2015;25(5):287-92.

115. Murphy PA, Avery M. Choosing Wisely for Health. Journal of Midwifery & Women's Health. 2015;60(3):235-6.

116. Mutter TC, Bryson GL. Choosing wisely and preoperative hemoglobin A1c testing: what should it mean? Canadian Journal of Anesthesia/Journal canadien d'anesthésie. 2016;63(12):1307-13.

117. Naik AD, Hinojosa-Lindsey M, Arney J, El-Serag HB, Hou J. Choosing Wisely and the Perceived Drivers of Endoscopy Use. Clinical Gastroenterology and Hepatology. 2013;11(7):753-5.

118. Naylor CD. The Complex World of Prescribing Behavior. JAMA. 2004;291(1):104-.

119. Neeman N, Quinn K, Soni K, Mourad M, Sehgal NL. Reducing Radiology Use on an Inpatient Medical Service: Choosing Wisely. Archives of Internal Medicine. 2012;172(20):1606-.

120. Niven DJ, Leigh JP, Stelfox HT. Ethical considerations in the de-adoption of ineffective or harmful aspects of healthcare. Healthcare Management Forum. 2016;29(5):214-7.

121. Noseworthy T, Clement F. Health technology reassessment: scope, methodology, & language. International Journal of Technology Assessment in Health Care. 2012;28(3):201-2.

122. Pandey A, Khera R, Kumar N, Golwala H, Girotra S, Fonarow GC. Use of Pulmonary Artery Catheterization in US Patients With Heart Failure, 2001-2012. JAMA Internal Medicine. 2016;176(1):129-.

123. Parkinson B, Sermet C, Clement F, Crausaz S, Godman B, Garner S, et al. Disinvestment and Value-Based Purchasing Strategies for Pharmaceuticals: An International Review. PharmacoEconomics. 2015;33(9):905-24.

124. Parks AL, O’Malley PG. From Choosing Wisely to Practicing Value—More to the Story. JAMA Internal Medicine. 2016;176(10):1571-.

125. Pearson S, Littlejohns P. Reallocating resources: how should the National Institute for Health and Clinical Excellence guide disinvestment efforts in the National Health Service? Journal of Health Services Research & Policy. 2007;12(3):160-5.

126. Pisetsky DS. The Choosing Wisely initiative: Does it have your back? Arthritis Research and Therapy. 2013;15(4):4-6.

127. Polisena J, Clifford T, Elshaug AG, Mitton C, Russell E, Skidmore B. Case studies that illustrate disinvestment and resource allocation decision-making processes in health care: a systematic review. Int J Technol Assess Health Care. 2013;29(2):174-84.

128. Prasad V, Ioannidis JPA. Evidence-based de-implementation for contradicted, unproven, and aspiring healthcare practices. Implementation Science. 2014;9(1):1-.

129. Raghavan D. Choosing Wisely: Where's the Beef? Journal of Oncology Practice. 2015;11(4):325-6.

130. Raman S, Chow R, Hoskin P, Chow E. How should radiation oncologists interpret the ASTRO evidence-based guideline and ASTRO Choosing Wisely campaign for the treatment of uncomplicated bone metastases? Practical Radiation Oncology. 2017;7(1):13-5.

131. Ramsey SD, Fedorenko C, Chauhan R, McGee R, Lyman GH, Kreizenbeck K, et al. Baseline Estimates of Adherence to American Society of Clinical Oncology/American Board of Internal Medicine Choosing Wisely Initiative Among Patients With Cancer Enrolled With a Large Regional Commercial Health Insurer. Journal of Oncology Practice. 2015;11(4):338-43.

132. Rao VM, Levin DC. The Overuse of Diagnostic Imaging and the Choosing Wisely Initiative. Annals of Internal Medicine. 2012;157(8):574-.

133. Rao VM, Levin DC. The Choosing Wisely Initiative of the American Board of Internal Medicine Foundation: What Will Its Impact Be on Radiology Practice? American Journal of Roentgenology. 2014;202(2):358-61.

134. Read AJ, Weissman A, Schoenfeld PS, Saini S, Menees SB, Saini SD. The Effect of Endoscopist Recommendations on PCPs Choosing Wisely about Colonoscopy. American Journal of Gastroenterology. 2016;111(5):749-51.

135. Resnick B, Fick DM. The Choosing Wisely® campaign and nurses role in dissemination. Geriatric Nursing. 2013;34(3):179-80.

136. Robert G, Harlock J, Williams I. Disentangling rhetoric and reality: an international Delphi study of factors and processes that facilitate the successful implementation of decisions to decommission healthcare services. Implementation Science. 2014;9(1):123-.

137. Rohren EM, Dillehay GL, Jadvar H. SNMMI Comment on ASCO 2013 "Choosing Wisely" Recommendation on Use of PET/CT in Recurrent Cancer Surveillance. Journal of Nuclear Medicine. 2014;55(5):699-700.

138. Roman BR, Asch DA. Faded Promises: The Challenge of Deadopting Low-Value Care. Annals of Internal Medicine. 2014;161(2):149-.

139. Rooshenas L, Owen-Smith A, Hollingworth W, Badrinath P, Beynon C, Donovan JL. "I won't call it rationing...": an ethnographic study of healthcare disinvestment in theory and practice. Soc Sci Med. 2015;128:273-81.

140. Rosenberg A, Agiro A, Gottlieb M, Barron J, Brady P, Liu Y, et al. Early Trends Among Seven Recommendations From the Choosing Wisely Campaign. JAMA Intern Med. 2015;175(12):1913-20.

141. Schwartz AL, Chernew ME, Landon BE, McWilliams JM. Changes in Low-Value Services in Year 1 of the Medicare Pioneer Accountable Care Organization Program. JAMA Internal Medicine. 2015;175(11):1815-.

142. Scott JW, Schwartz AL, Gates JD, Gerhard‐Herman M, Havens JM. Choosing Wisely for Syncope: Low‐Value Carotid Ultrasound Use. Journal of the American Heart Association. 2014;3(4):1-8.

143. Sharp AL, Klau MH, Keschner D, Macy E, Tang T, Shen E, et al. Low-value care for acute sinusitis encounters: Who's choosing wisely. American Journal of Managed Care. 2015;21(7):479-85.

144. Silverstein W, Lass E, Born K, Morinville A, Levinson W, Tannenbaum C. A survey of primary care patients’ readiness to engage in the de-adoption practices recommended by Choosing Wisely Canada. BMC Research Notes. 2016;9(1):301-.

145. Simos D, Hutton B, Clemons M. Are Physicians Choosing Wisely When Imaging for Distant Metastases in Women With Operable Breast Cancer? Journal of Oncology Practice. 2015;11(1):62-8.

146. Siontis GCM, Tatsioni A, Katritsis DG, Ioannidis JPA. Persistent reservations against contradicted percutaneous coronary intervention indications: Citation content analysis. American Heart Journal. 2009;157(4):695-701.

147. Siwek J, Lin KW. More ways to improve health and reduce harm: choosing wisely phase 3. American family physician. 2014;89(5):329-.

148. Sorrel ALy. Choosing wisely. Texas medicine. 2012;108(12):25-.

149. Speight J, Browne JL, Furler JS. Testing times! Choosing Wisely when it comes to monitoring type 2 diabetes. Medical Journal of Australia. 2015;203(9):354-6.e1.

150. Stafford RS. Impact of Clinical Trial Results on National Trends in α-Blocker Prescribing, 1996-2002. JAMA. 2004;291(1):54-.

151. Street JM, Hennessy SE, Watt AM, Hiller JE, Elshaug AG. News and social media: Windows into community perspectives on disinvestment. International Journal of Technology Assessment in Health Care. 2011;27(4):376-83.

152. Sukel MPP, van der Linden MW, Chen C, Erkens JA, Herings RMC. Large-scale stopping and switching treatment with COX-2 inhibitors after the rofecoxib withdrawal. Pharmacoepidemiology and Drug Safety. 2008;17(1):9-19.

153. Surial B, Burkhart A, Terliesner N, Morgenthaler M, Bächli E. Adherence to transfusion guidelines: are we prepared for the Smarter Medicine or Choosing Wisely® initiative? Swiss Medical Weekly. 2015;145(January):1-8.

154. Tanaka LA, Khan OA, Jackson EV, Miller KE, Chiam TC. Choosing Wisely in Delaware: Rationale for Evidence-Based Diagnosis & Evaluation of Low Back Pain. Delaware medical journal. 2016;88(1):14-8.

155. Tatsioni A, Bonitsis NG, Ioannidis JPA. Persistence of Contradicted Claims in the Literature. JAMA. 2007;298(21):2517-.

156. Tatsioni A, Siontis GCM, Ioannidis JPA. Partisan Perspectives in the Medical Literature: A Study of High Frequency Editorialists Favoring Hormone Replacement Therapy. Journal of General Internal Medicine. 2010;25(9):914-9.

157. Thiebaud P, Patel BV, Nichol MB. Impact of Rofecoxib Withdrawal on Cyclooxygenase-2 Utilization among Patients with and without Cardiovascular Risk. Value in Health. 2006;9(6):361-8.

158. Urness D, Parker NJ, Rapoport MJ, Wilkes TCR. Choosing Wisely. The Canadian Journal of Psychiatry. 2016;61(11):700-4.

159. Vogel L. Choosing Wisely Canada seeks system change. Canadian Medical Association Journal. 2016;188(8):E135-E6.

160. Vogel L. Choosing Wisely around the world. Canadian Medical Association Journal. 2015;187(11):E341-E2.

161. Vogel L. More hospitals Choosing Wisely. Canadian Medical Association Journal. 2015;187(10):722-.

162. Volpp KG, Loewenstein G, Asch DA. Choosing wisely: Low-value services, utilization, and patient cost sharing. 2012. p. 1635-6.

163. Voorn VMA, Marang-van de Mheen PJ, So-Osman C, Vliet Vlieland TPM, Koopman-van Gemert AWMM, Nelissen RGHH, et al. Designing a strategy to implement cost-effective blood transfusion management in elective hip and knee arthroplasties: A study protocol. Implementation Science. 2012;7(1):58-.

164. Voorn VMA, Marang-van de Mheen PJ, So-Osman C, Kaptein AA, van der Hout A, van den Akker-van Marle ME, et al. De-implementation of expensive blood saving measures in hip and knee arthroplasties: study protocol for the LISBOA-II cluster randomized trial. Implementation Science. 2014;9(1):48-.

165. Wang MTM, Gamble G, Grey A. Responses of Specialist Societies to Evidence for Reversal of Practice. JAMA Internal Medicine. 2015;175(5):845-.

166. Watt AM, Hiller JE, Braunack-Mayer AJ, Moss JR, Buchan H, Wale J, et al. The ASTUTE Health study protocol: Deliberative stakeholder engagements to inform implementation approaches to healthcare disinvestment. Implementation Science. 2012;7(1):101.

167. Watt AM, Willis CD, Hodgetts K, Elshaug AG, Hiller JE. Engaging clinicians in evidence-based disinvestment: role and perceptions of evidence. International Journal of Technology Assessment in Health Care. 2012;28(3):211-9.

168. Wolfson D, Suchman A. Choosing Wisely®: A case study of constructive engagement in health policy. Healthcare. 2016;4(3):240-3.

169. Wolfson DB. Choosing Wisely campaign builds momentum. American Journal of Managed Care. 2016;22(7):495-6.

170. Wolfson D, Santa J, Slass L. Engaging Physicians and Consumers in Conversations About Treatment Overuse and Waste. Academic Medicine. 2014;89(7):990-5.

171. Wong CJ, Gaster B, Dugdale DC. Choosing Wisely. American Journal of Preventive Medicine. 2014;47(5):653-5.

172. Zikmund-Fisher BJ, Kullgren JT, Fagerlin A, Klamerus ML, Bernstein SJ, Kerr EA. Perceived Barriers to Implementing Individual Choosing Wisely® Recommendations in Two National Surveys of Primary Care Providers. Journal of General Internal Medicine. 2017;32(2):210-7.
