# Supplementary material for: Determinants of the de-implementation of low-value care: a multi-method study
Source: BMC Health Serv Res. 2022 Apr 6;22:450. doi: 10.1186/s12913-022-07827-4 (PMC8985316; doi:10.1186/s12913-022-07827-4)
Supplement: Supplementary file 5 — Additional file 5. Quality assessments of included original research articles (n = 74) [file 12913_2022_7827_MOESM5_ESM.docx]

| **Author, year**  **Additional File 5. Quality assessments of included original research articles^a,b^ (n=74)** | **Explicit theoretical framework** | **Statement of aims/objectives in main body of report** | **Clear description of research setting** | **Evidence of sample size considered in terms of analysis** | **Representative sample of target group of a reasonable size** | **Description of procedure for data collection** | **Rationale for choice of data collection tool(s)** | **Detailed recruitment data** | **Statistical assessment of reliability and validity of measurement tool(s)^c^** | **Fit between stated research question and method of data collection^c^** | **Fit between stated research question and format and content of data collection tool(s)^d^** | **Fit between research question and method of analysis** | **Good justification for analytical method selected** | **Assessment of reliability of analytical process^d^** | **Evidence of user involvement in design** | **Strengths and limitations critically discussed** | **Sum** | | **% Score** |
| --- | --- | --- | --- | --- | --- | --- | --- | --- | --- | --- | --- | --- | --- | --- | --- | --- | --- | --- | --- |
| **Qualitative studies (scored out of 42)** | | | | | | | | | | | | | | | | | | | |
| Elshaug(1), 2008 | 1 | 1 | 3 | 3 | 2 | 3 | 0 | 2 | n/a | n/a | 3 | 3 | 0 | 0 | 0 | 0 | 18 | 43% | |
| Henshall(2), 2012 | 1 | 0 | 2 | 0 | 2 | 0 | 0 | 0 | n/a | n/a | 2 | 2 | 0 | 0 | 0 | 0 | 7 | 17% | |
| Hislop(3), 2011 | 0 | 3 | 2 | 0 | 1 | 0 | 0 | 0 | n/a | n/a | 3 | 3 | 0 | 0 | 0 | 1 | 10 | 24% | |
| Hodgetts(4), 2012 | 3 | 2 | 3 | 0 | 2 | 1 | 2 | 1 | n/a | n/a | 2 | 2 | 3 | 0 | 0 | 0 | 19 | 45% | |
| Horvath(5), 2016 | 2 | 3 | 3 | 0 | 2 | 3 | 3 | 3 | n/a | n/a | 3 | 3 | 3 | 1 | 0 | 3 | 28 | 67% | |
| Ibargoyen-Roteta(6), 2010 | 2 | 2 | 3 | 0 | 2 | 2 | 1 | 0 | n/a | n/a | 3 | 3 | 1 | 3 | 3 | 3 | 22 | 52% | |
| MacKean(7), 2013 | 1 | 2 | 1 | 0 | 1 | 1 | 0 | 0 | n/a | n/a | 2 | 3 | 0 | 0 | 0 | 0 | 9 | 21% | |
| Naik(8), 2013 | 2 | 1 | 2 | 3 | 2 | 2 | 1 | 0 | n/a | n/a | 3 | 3 | 0 | 0 | 0 | 0 | 16 | 38% | |
| Rooshenas(9), 2015 | 3 | 3 | 2 | 2 | 2 | 3 | 3 | 2 | n/a | n/a | 3 | 3 | 2 | 2 | 0 | 3 | 28 | 67% | |
| Siontis(10), 2009 | 1 | 3 | 2 | 2 | 3 | 2 | 1 | 2 | n/a | n/a | 2 | 2 | 0 | 1 | 0 | 2 | 20 | 48% | |
| Street(11),2011 | 3 | 3 | 3 | 1 | 2 | 2 | 2 | 3 | n/a | n/a | 3 | 3 | 1 | 1 | 0 | 1 | 24 | 57% | |
| Watt(12), 2012 | 2 | 2 | 3 | 2 | 2 | 3 | 3 | 3 | n/a | n/a | 3 | 3 | 2 | 1 | 0 | 0 | 25 | 60% | |
| Watt(13), 2012 | 3 | 2 | 2 | 0 | 2 | 3 | 1 | 1 | n/a | n/a | 3 | 3 | 0 | 0 | 2 | 0 | 19 | 45% | |
| **Quantitative studies (scored out of 42)** | | | | | | | | | | | | | | | | | | | |
| Atwater(14), 2009 | 0 | 2 | 3 | 1 | 2 | 1 | 0 | 3 | 0 | 3 | n/a | 3 | 1 | n/a | 0 | 1 | 17 | 40% | |
| Balekian(15), 2016 | 2 | 2 | 2 | 1 | 2 | 1 | 1 | 3 | 0 | 2 | n/a | 3 | 2 | n/a | 0 | 1 | 20 | 48% | |
| Brunt(16), 2003 | 0 | 2 | 3 | 1 | 2 | 2 | 3 | 2 | 2 | 2 | n/a | 2 | 3 | n/a | 0 | 3 | 23 | 55% | |
| Colla(17), 2016 | 2 | 2 | 2 | 1 | 2 | 3 | 2 | 2 | 3 | 3 | n/a | 3 | 3 | n/a | 0 | 2 | 24 | 57% | |
| Colla(18), 2015 | 3 | 2 | 2 | 1 | 2 | 3 | 3 | 3 | 1 | 3 | n/a | 3 | 3 | n/a | 1 | 2 | 28 | 67% | |
| Colla(19), 2015 | 2 | 3 | 1 | 2 | 3 | 2 | 1 | 2 | 2 | 3 | n/a | 3 | 2 | n/a | 0 | 1 | 22 | 52% | |
| Deyell(20), 2011 | 1 | 2 | 2 | 3 | 2 | 3 | 3 | 3 | 2 | 3 | n/a | 3 | 2 | n/a | 0 | 3 | 27 | 64% | |
| Edwards(21), 2014 | 3 | 1 | 2 | 1 | 2 | 3 | 3 | 1 | 0 | 2 | n/a | 2 | 2 | n/a | 0 | 3 | 23 | 55% | |
| Ferrari(22), 2015 | 2 | 3 | 3 | 3 | 2 | 3 | 2 | 3 | 0 | 3 | n/a | 3 | 2 | n/a | 0 | 2 | 28 | 67% | |
| Gerdvilaite(23), 2011 | 1 | 2 | 1 | 2 | 2 | 3 | 2 | 3 | 1 | 3 | n/a | 3 | 1 | n/a | 0 | 1 | 25 | 60% | |
| Gershengorn(24), 2013 | 0 | 3 | 3 | 2 | 2 | 2 | 0 | 0 | 0 | 3 | n/a | 3 | 2 | n/a | 0 | 3 | 20 | 48% | |
| Gidwani(25), 2016 | 1 | 1 | 2 | 0 | 3 | 3 | 3 | 2 | 0 | 3 | n/a | 3 | 2 | n/a | 0 | 2 | 22 | 52% | |
| Greene(26), 2015 | 1 | 1 | 1 | 0 | 2 | 1 | 0 | 2 | 0 | 2 | n/a | 3 | 0 | n/a | 0 | 0 | 11 | 26% | |
| Haas(27), 2004 | 0 | 3 | 2 | 0 | 1 | 3 | 1 | 3 | 0 | 2 | n/a | 3 | 3 | n/a | 0 | 2 | 21 | 50% | |
| Hauptman(28), 2006 | 1 | 1 | 2 | 0 | 3 | 3 | 2 | 3 | 0 | 3 | n/a | 3 | 2 | n/a | 0 | 3 | 23 | 55% | |
| Hersh(29), 2004 | 1 | 1 | 3 | 0 | 2 | 3 | 2 | 2 | 3 | 2 | n/a | 3 | 2 | n/a | 0 | 2 | 21 | 50% | |
| Hicks(30), 2016 | 1 | 1 | 3 | 0 | 2 | 3 | 2 | 3 | 1 | 3 | n/a | 3 | 1 | n/a | 1 | 0 | 20 | 48% | |
| Hines(31), 2015 | 2 | 1 | 2 | 0 | 1 | 2 | 2 | 2 | 0 | 2 | n/a | 2 | 1 | n/a | 0 | 3 | 18 | 43% | |
| Howard(32), 2011 | 1 | 2 | 2 | 0 | 2 | 2 | 1 | 0 | 0 | 3 | n/a | 2 | 1 | n/a | 0 | 2 | 15 | 36% | |
| Howard(33), 2012 | 2 | 3 | 3 | 1 | 2 | 2 | 3 | 1 | n/a | 3 | n/a | 3 | 2 | n/a | 0 | 1 | 26 | 67% | |
| Hsiao(34), 2009 | 1 | 1 | 3 | 0 | 2 | 2 | 2 | 2 | 0 | 3 | n/a | 3 | 3 | n/a | 0 | 3 | 22 | 52% | |
| Huang(35), 2007 | 2 | 2 | 2 | 2 | 3 | 3 | 3 | 3 | 1 | 3 | n/a | 3 | 3 | n/a | 0 | 3 | 29 | 69% | |
| Ioannidis(36), 2005 | 2 | 1 | 3 | 2 | 2 | 3 | 3 | 3 | 3 | 2 | n/a | 3 | 2 | n/a | 0 | 2 | 26 | 62% | |
| Kahn(37), 2016 | 2 | 1 | 2 | 1 | 2 | 3 | 1 | 1 | 2 | 3 | n/a | 2 | 2 | n/a | 0 | 3 | 20 | 48% | |
| Koo(38), 2011 | 2 | 2 | 3 | 1 | 3 | 3 | 3 | 3 | 3 | 3 | n/a | 3 | 2 | n/a | 1 | 2 | 28 | 67% | |
| Kost(39), 2015 | 1 | 1 | 2 | 1 | 2 | 1 | 1 | 1 | 0 | 3 | n/a | 2 | 0 | n/a | 0 | 1 | 13 | 31% | |
| Krol(40), 2004 | 0 | 3 | 3 | 1 | 2 | 3 | 2 | 2 | 2 | 3 | n/a | 3 | 1 | n/a | 0 | 1 | 21 | 50% | |
| Kulawik(41), 2009 | 0 | 1 | 1 | 1 | 3 | 1 | 0 | 1 | 0 | 3 | n/a | 3 | 1 | n/a | 0 | 0 | 12 | 29% | |
| Lasser(42), 2016 | 1 | 2 | 2 | 1 | 2 | 2 | 2 | 2 | 0 | 3 | n/a | 3 | 2 | n/a | 0 | 2 | 21 | 50% | |
| Lawton(43), 2003 | 1 | 1 | 2 | 1 | 2 | 2 | 1 | 3 | 0 | 2 | n/a | 3 | 1 | n/a | 0 | 1 | 18 | 43% | |
| Leggett(44), 2012 | 1 | 3 | 1 | 2 | 2 | 3 | 2 | 3 | 1 | 3 | n/a | 3 | 1 | n/a | 0 | 2 | 27 | 64% | |
| Lesuis(45), 2016 | 2 | 1 | 3 | 0 | 2 | 3 | 1 | 3 | 2 | 2 | n/a | 2 | 3 | n/a | 0 | 3 | 23 | 55% | |
| Majumdar(46), 2004 | 2 | 1 | 3 | 0 | 3 | 3 | 3 | 2 | 0 | 2 | n/a | 2 | 2 | n/a | 0 | 3 | 24 | 57% | |
| Makarov(47), 2015 | 2 | 2 | 2 | 1 | 2 | 2 | 2 | 2 | 0 | 3 | n/a | 3 | 3 | n/a | 0 | 3 | 24 | 57% | |
| Mnatzaganian(48), 2015 | 2 | 1 | 3 | 0 | 2 | 3 | 3 | 1 | 3 | 2 | n/a | 2 | 2 | n/a | 0 | 2 | 21 | 50% | |
| Murphy(49), 2013 | 1 | 2 | 3 | 0 | 2 | 2 | 2 | 0 | 3 | 3 | n/a | 2 | 3 | n/a | 0 | 3 | 20 | 48% | |
| Neeman(50), 2012 | 1 | 1 | 1 | 0 | 1 | 2 | 0 | 1 | 0 | 2 | n/a | 2 | 1 | n/a | 0 | 0 | 10 | 24% | |
| Pandey(51), 2016 | 1 | 1 | 1 | 1 | 3 | 1 | 1 | 1 | 2 | 2 | n/a | 3 | 0 | n/a | 0 | 0 | 13 | 31% | |
| Polisena(52), 2013 | 0 | 2 | 1 | 2 | 2 | 2 | 1 | 3 | 1 | 3 | n/a | 3 | 1 | n/a | 0 | 3 | 24 | 57% | |
| Ramsey(53), 2015 | 1 | 2 | 3 | 2 | 2 | 2 | 3 | 2 | 0 | 3 | n/a | 2 | 2 | n/a | 0 | 2 | 23 | 55% | |
| Rosenberg(54), 2015 | 2 | 2 | 2 | 1 | 3 | 3 | 3 | 3 | 2 | 3 | n/a | 3 | 3 | n/a | 0 | 2 | 27 | 64% | |
| Schwartz(55), 2015 | 2 | 2 | 3 | 1 | 3 | 3 | 3 | 3 | 1 | 3 | n/a | 3 | 3 | n/a | 0 | 2 | 28 | 67% | |
| Scott(56), 2014 | 2 | 3 | 2 | 2 | 2 | 3 | 2 | 3 | 1 | 2 | n/a | 2 | 2 | n/a | 0 | 2 | 25 | 60% | |
| Sharp(57), 2015 | 2 | 3 | 3 | 2 | 3 | 3 | 2 | 3 | 3 | 3 | n/a | 3 | 2 | n/a | 0 | 3 | 29 | 69% | |
| Simos(58), 2015 | 2 | 2 | 1 | 0 | 2 | 2 | 1 | 2 | 0 | 2 | n/a | 2 | 2 | n/a | 0 | 2 | 18 | 43% | |
| Stafford(59), 2004 | 2 | 2 | 2 | 0 | 2 | 2 | 3 | 1 | 3 | 3 | n/a | 3 | 3 | n/a | 0 | 2 | 22 | 52% | |
| Sukel(60), 2008 | 2 | 3 | 3 | 0 | 3 | 3 | 2 | 3 | 0 | 3 | n/a | 3 | 2 | n/a | 0 | 2 | 26 | 62% | |
| Surial(61), 2015 | 2 | 3 | 3 | 0 | 2 | 2 | 2 | 2 | 0 | 3 | n/a | 3 | 1 | n/a | 0 | 0 | 20 | 48% | |
| Tatsioni(62), 2010 | 0 | 3 | 3 | 1 | 2 | 3 | 1 | 3 | 0 | 2 | n/a | 2 | 0 | n/a | 0 | 0 | 18 | 43% | |
| Thiebaud(63), 2006 | 3 | 1 | 2 | 0 | 3 | 3 | 2 | 3 | 0 | 3 | n/a | 3 | 2 | n/a | 0 | 2 | 24 | 57% | |
| Wang(64), 2015 | 1 | 1 | 1 | 0 | 2 | 2 | 0 | 1 | 2 | 3 | n/a | 3 | 0 | n/a | 0 | 0 | 11 | 26% | |
| Zikmund-Fisher(65), 2017 | 2 | 3 | 2 | 0 | 2 | 2 | 1 | 3 | 0 | 3 | n/a | 3 | 1 | n/a | 2 | 2 | 23 | 55% | |
| **Mixed-methods studies (scored out of 48)** | | | | | | | | | | | | | | | | | | | |
| Azermai(66), 2014 | 1 | 2 | 2 | 1 | 2 | 3 | 1 | 2 | 0 | 3 | 2 | 3 | 2 | 0 | 2 | 3 | 24 | 50% | |
| Hollingworth(67), 2015 | 3 | 3 | 3 | 3 | 3 | 3 | 3 | 2 | 3 | 3 | 2 | 3 | 3 | 3 | 2 | 3 | 34 | 71% | |
| Ibargoyen-Roteta(68), 2009 | 1 | 3 | 2 | 0 | 2 | 2 | 2 | 1 | 0 | 2 | 2 | 3 | 2 | 0 | 0 | 1 | 19 | 40% | |
| Leggett(69), 2012 | 2 | 3 | 1 | 2 | 3 | 2 | 2 | 3 | 0 | 3 | 2 | 3 | 0 | 0 | 0 | 1 | 22 | 46% | |
| Lesuis(70), 2017 | 1 | 1 | 2 | 1 | 2 | 1 | 1 | 2 | 0 | 3 | 3 | 3 | 2 | 0 | 0 | 1 | 17 | 35% | |
| Massatti(71), 2008 | 2 | 1 | 2 | 0 | 3 | 3 | 3 | 0 | 1 | 3 | 3 | 3 | 3 | 3 | 0 | 2 | 22 | 46% | |
| Robert(72), 2014 | 3 | 3 | 2 | 2 | 2 | 3 | 3 | 3 | 2 | 3 | 3 | 3 | 1 | 0 | 0 | 3 | 28 | 58% | |
| Silverstein(73), 2016 | 3 | 3 | 2 | 0 | 2 | 3 | 3 | 3 | 2 | 3 | 2 | 3 | 3 | 2 | 0 | 3 | 28 | 58% | |
| Tatsioni(74), 2007 | 0 | 2 | 2 | 2 | 3 | 2 | 1 | 2 | 0 | 3 | 3 | 3 | 2 | 0 | 0 | 0 | 19 | 40% | |

Legend

1. 74 of 76 original research articles assessed for quality as 2 articles(75, 76) reported on study protocols that were not appropriate to assess for study quality.
2. For each quality assessment criterion, the score ranged from 0-3, with 14 of 16 criteria applicable to qualitative studies, 14 of 16 for quantitative studies, and all 16 to mixed methods studies.
3. Criteria apply to quantitative studies
4. Criteria apply to qualitative studies

**References**

1. Elshaug AG, Hiller JE, Moss JR. Exploring policy-makers' perspectives on disinvestment from ineffective healthcare practices. 2008. p. 1-9.

2. Henshall C, Schuller T, Mardhani-Bayne L. Using health technology assessment to support optimal use of technologies in current practice: the challenge of “disinvestment”. International Journal of Technology Assessment in Health Care. 2012;28(3):203-10.

3. Hislop JM. PHP129 Societal Preferences for Health Technology Disinvestment Policy: Views of Scottish Taxpayers &#x2013; A Qualitative Study. Value in Health. 2011;14(7):A356-A7.

4. Hodgetts K, Elshaug AG, Hiller JE. What counts and how to count it: Physicians’ constructions of evidence in a disinvestment context. Social Science & Medicine. 2012;75(12):2191-9.

5. Horvath K, Semlitsch T, Jeitler K, Abuzahra ME, Posch N, Domke A, et al. Choosing Wisely: assessment of current US top five list recommendations’ trustworthiness using a pragmatic approach. BMJ Open. 2016;6(10):e012366-e.

6. Ibargoyen-Roteta N, Gutiérrez-Ibarluzea I, Asua J. Guiding the process of health technology disinvestment. Health Policy. 2010;98(2-3):218-26.

7. MacKean G, Noseworthy T, Elshaug AG, Leggett L, Littlejohns P, Berezanski J, et al. Health technology reassessment: the art of the possible. International Journal of Technology Assessment in Health Care. 2013;29(4):418-23.

8. Naik AD, Hinojosa-Lindsey M, Arney J, El-Serag HB, Hou J. Choosing Wisely and the Perceived Drivers of Endoscopy Use. Clinical Gastroenterology and Hepatology. 2013;11(7):753-5.

9. Rooshenas L, Owen-Smith A, Hollingworth W, Badrinath P, Beynon C, Donovan JL. "I won't call it rationing...": an ethnographic study of healthcare disinvestment in theory and practice. Soc Sci Med. 2015;128:273-81.

10. Siontis GCM, Tatsioni A, Katritsis DG, Ioannidis JPA. Persistent reservations against contradicted percutaneous coronary intervention indications: Citation content analysis. American Heart Journal. 2009;157(4):695-701.

11. Street JM, Hennessy SE, Watt AM, Hiller JE, Elshaug AG. News and social media: Windows into community perspectives on disinvestment. International Journal of Technology Assessment in Health Care. 2011;27(4):376-83.

12. Watt AM, Willis CD, Hodgetts K, Elshaug AG, Hiller JE. Engaging clinicians in evidence-based disinvestment: role and perceptions of evidence. International Journal of Technology Assessment in Health Care. 2012;28(3):211-9.

13. Watt AM, Hiller JE, Braunack-Mayer AJ, Moss JR, Buchan H, Wale J, et al. The ASTUTE Health study protocol: Deliberative stakeholder engagements to inform implementation approaches to healthcare disinvestment. Implementation Science. 2012;7(1):101.

14. Atwater BD, Oujiri J, Wolff MR. The immediate impact of the Clinical Outcomes Utilizing Revascularization and Aggressive Drug Evaluation (COURAGE) trial on the management of stable angina. Clin Cardiol. 2009;32(8):E1-3.

15. Balekian AA, Fisher JM, Gould MK. Brain Imaging for Staging of Patients With Clinical Stage IA Non-small Cell Lung Cancer in the National Lung Screening Trial. Chest. 2016;149(4):943-50.

16. Brunt ME, Murray MD, Hui SL, Kesterson J, Perkins AJ, Tierney WM. Mass media release of medical research results: an analysis of antihypertensive drug prescribing in the aftermath of the calcium channel blocker scare of March 1995. J Gen Intern Med. 2003;18(2):84-94.

17. Colla CH, Kinsella EA, Morden NE, Meyers DJ, Rosenthal MB, Sequist TD. Physician perceptions of Choosing Wisely and drivers of overuse. The American journal of managed care. 2016;22(5):337-43.

18. Colla CH, Sequist TD, Rosenthal MB, Schpero WL, Gottlieb DJ, Morden NE. Use of non-indicated cardiac testing in low-risk patients: Choosing Wisely. BMJ Quality & Safety. 2015;24(2):149-53.

19. Colla CH, Morden NE, Sequist TD, Schpero WL, Rosenthal MB. Choosing Wisely: Prevalence and Correlates of Low-Value Health Care Services in the United States. Journal of General Internal Medicine. 2015;30(2):221-8.

20. Deyell MW, Buller CE, Miller LH, Wang TY, Dai D, Lamas GA, et al. Impact of National Clinical Guideline recommendations for revascularization of persistently occluded infarct-related arteries on clinical practice in the United States. Arch Intern Med. 2011;171(18):1636-43.

21. Edwards RT, Charles JM, Thomas S, Bishop J, Cohen D, Groves S, et al. A national Programme Budgeting and Marginal Analysis (PBMA) of health improvement spending across Wales: disinvestment and reinvestment across the life course. BMC Public Health. 2014;14(1):837-.

22. Ferrari R. Evaluation of the Canadian Rheumatology Association Choosing Wisely recommendation concerning anti-nuclear antibody (ANA) testing. Clinical Rheumatology. 2015;34(9):1551-6.

23. Gerdvilaite J, Nachtnebel A, editors. Disinvestment. Overview of disinvestment experiences and challenges in selected countries2011.

24. Gershengorn HB, Wunsch H. Understanding Changes in Established Practice. Critical Care Medicine. 2013;41(12):2667-76.

25. Gidwani R, Sinnott P, Avoundjian T, Lo J, Asch SM, Barnett PG. Inappropriate ordering of lumbar spine magnetic resonance imaging: are providers Choosing Wisely? The American journal of managed care. 2016;22(2):e68-76.

26. Greene SE, Massone R. A survey of emergency medicine residents’ perspectives of the choosing wisely campaign. The American Journal of Emergency Medicine. 2015;33(6):853-5.

27. Haas JS, Kaplan CP, Gerstenberger EP, Kerlikowske K. Changes in the Use of Postmenopausal Hormone Therapy after the Publication of Clinical Trial Results. Annals of Internal Medicine. 2004;140(3):184-.

28. Hauptman PJ, Schnitzler MA, Swindle J, Burroughs TE. Use of Nesiritide Before and After Publications Suggesting Drug-Related Risks in Patients With Acute Decompensated Heart Failure. JAMA. 2006;296(15):1877-.

29. Hersh AL, Stefanick ML, Stafford RS. National use of postmenopausal hormone therapy. ACC Current Journal Review. 2004;13(4):18-.

30. Hicks LK, Rajasekhar A, Bering H, Carson KR, Kleinerman J, Kukreti V, et al. Identifying existing Choosing Wisely recommendations of high relevance and importance to hematology. American Journal of Hematology. 2016;91(8):787-92.

31. Hines JZ, Sewell JL, Sehgal NL, Moriates C, Horton CK, Chen AH. “Choosing Wisely” in an Academic Department of Medicine. American Journal of Medical Quality. 2015;30(6):566-70.

32. Howard DH, Kenline C, Lazarus HM, LeMaistre CF, Maziarz RT, McCarthy Jr PL, et al. Abandonment of High-Dose Chemotherapy/Hematopoietic Cell Transplants for Breast Cancer Following Negative Trial Results. Health Services Research. 2011;46(6pt1):1762-77.

33. Howard DH, Shen Y-C. Comparative Effectiveness Research, Technological Abandonment, and Health Care Spending. 23: Emerald Group Publishing Ltd.; 2012. p. 103-21.

34. Hsiao F-Y, Tsai Y-W, Huang W-F. Changes in physicians' practice of prescribing cyclooxygenase-2 inhibitor after market withdrawal of rofecoxib: A retrospective study of physician-patient pairs in Taiwan. Clinical Therapeutics. 2009;31(11):2618-27.

35. Huang W-F, Tsai Y-W, Hsiao F-Y, Liu W-C. Changes of the prescription of hormone therapy in menopausal women: An observational study in Taiwan. BMC Public Health. 2007;7(1):56-.

36. Ioannidis JPA. Contradicted and Initially Stronger Effects in Highly Cited Clinical Research. JAMA. 2005;294(2):218-.

37. Kahn JM, Le TQ. Adoption and de-adoption of drotrecogin alfa for severe sepsis in the United States. Journal of Critical Care. 2016;32:114-9.

38. Koo KKY, Sun JCJ, Zhou Q, Guyatt G, Cook DJ, Walter SD, et al. Pulmonary artery catheters: Evolving rates and reasons for use*. Critical Care Medicine. 2011;39(7):1613-8.

39. Kost A, Genao I, Lee JW, Smith SR. Clinical Decisions Made in Primary Care Clinics Before and After Choosing Wisely. The Journal of the American Board of Family Medicine. 2015;28(4):471-4.

40. Krol N, Wensing M, Haaijer-Ruskamp F, Muris JWM, Numans ME, Schattenberg G, et al. Patient-directed strategy to reduce prescribing for patients with dyspepsia in general practice: a randomized trial. Alimentary Pharmacology and Therapeutics. 2004;19(8):917-22.

41. Kulawik D, Sands JJ, Mayo K, Fenderson M, Hutchinson J, Woodward C, et al. Focused Vascular Access Education to Reduce the Use of Chronic Tunneled Hemodialysis Catheters: Results of a Network Quality Improvement Initiative. Seminars in Dialysis. 2009;22(6):692-7.

42. Lasser EC, Pfoh ER, Chang HY, Chan KS, Bailey JC, Kharrazi H, et al. Has Choosing Wisely® affected rates of dual-energy X-ray absorptiometry use? Osteoporosis International. 2016;27(7):2311-6.

43. Lawton B. Changes in use of hormone replacement therapy after the report from the Women's Health Initiative: cross sectional survey of users. BMJ. 2003;327(7419):845-6.

44. Leggett L, Noseworthy TW, Zarrabi M, Lorenzetti D, Sutherland LR, Clement FM. HEALTH TECHNOLOGY REASSESSMENT OF NON-DRUG TECHNOLOGIES: CURRENT PRACTICES. International Journal of Technology Assessment in Health Care. 2012;28(3):220-7.

45. Lesuis N, Hulscher MEJL, Piek E, Demirel H, van der Laan-Baalbergen N, Meek I, et al. Choosing Wisely in Daily Practice: An Intervention Study on Antinuclear Antibody Testing by Rheumatologists. Arthritis Care & Research. 2016;68(4):562-9.

46. Majumdar SR. Promotion and Prescribing of Hormone Therapy After Report of Harm by the Women’s Health Initiative. JAMA. 2004;292(16):1983-.

47. Makarov DV, Soulos PR, Gold HT, Yu JB, Sen S, Ross JS, et al. Regional-Level Correlations in Inappropriate Imaging Rates for Prostate and Breast Cancers. JAMA Oncology. 2015;1(2):185-.

48. Mnatzaganian G, Karnon J, Moss JR, Elshaug AG, Metz M, Frank OR, et al. Informing disinvestment with limited evidence: cobalamin deficiency in the fatigued. International Journal of Technology Assessment in Health Care. 2015;31(3):188-96.

49. Murphy DJ, Needham DM, Netzer G, Zeger SL, Colantuoni E, Ness P, et al. RBC Transfusion Practices Among Critically Ill Patients. Critical Care Medicine. 2013;41(10):2344-53.

50. Neeman N, Quinn K, Soni K, Mourad M, Sehgal NL. Reducing Radiology Use on an Inpatient Medical Service: Choosing Wisely. Archives of Internal Medicine. 2012;172(20):1606-.

51. Pandey A, Khera R, Kumar N, Golwala H, Girotra S, Fonarow GC. Use of Pulmonary Artery Catheterization in US Patients With Heart Failure, 2001-2012. JAMA Internal Medicine. 2016;176(1):129-.

52. Polisena J, Clifford T, Elshaug AG, Mitton C, Russell E, Skidmore B. Case studies that illustrate disinvestment and resource allocation decision-making processes in health care: a systematic review. Int J Technol Assess Health Care. 2013;29(2):174-84.

53. Ramsey SD, Fedorenko C, Chauhan R, McGee R, Lyman GH, Kreizenbeck K, et al. Baseline Estimates of Adherence to American Society of Clinical Oncology/American Board of Internal Medicine Choosing Wisely Initiative Among Patients With Cancer Enrolled With a Large Regional Commercial Health Insurer. Journal of Oncology Practice. 2015;11(4):338-43.

54. Rosenberg A, Agiro A, Gottlieb M, Barron J, Brady P, Liu Y, et al. Early Trends Among Seven Recommendations From the Choosing Wisely Campaign. JAMA Intern Med. 2015;175(12):1913-20.

55. Schwartz AL, Chernew ME, Landon BE, McWilliams JM. Changes in Low-Value Services in Year 1 of the Medicare Pioneer Accountable Care Organization Program. JAMA Internal Medicine. 2015;175(11):1815-.

56. Scott JW, Schwartz AL, Gates JD, Gerhard‐Herman M, Havens JM. Choosing Wisely for Syncope: Low‐Value Carotid Ultrasound Use. Journal of the American Heart Association. 2014;3(4):1-8.

57. Sharp AL, Klau MH, Keschner D, Macy E, Tang T, Shen E, et al. Low-value care for acute sinusitis encounters: Who's choosing wisely. American Journal of Managed Care. 2015;21(7):479-85.

58. Simos D, Hutton B, Clemons M. Are Physicians Choosing Wisely When Imaging for Distant Metastases in Women With Operable Breast Cancer? Journal of Oncology Practice. 2015;11(1):62-8.

59. Stafford RS. Impact of Clinical Trial Results on National Trends in α-Blocker Prescribing, 1996-2002. JAMA. 2004;291(1):54-.

60. Sukel MPP, van der Linden MW, Chen C, Erkens JA, Herings RMC. Large-scale stopping and switching treatment with COX-2 inhibitors after the rofecoxib withdrawal. Pharmacoepidemiology and Drug Safety. 2008;17(1):9-19.

61. Surial B, Burkhart A, Terliesner N, Morgenthaler M, Bächli E. Adherence to transfusion guidelines: are we prepared for the Smarter Medicine or Choosing Wisely® initiative? Swiss Medical Weekly. 2015;145(January):1-8.

62. Tatsioni A, Siontis GCM, Ioannidis JPA. Partisan Perspectives in the Medical Literature: A Study of High Frequency Editorialists Favoring Hormone Replacement Therapy. Journal of General Internal Medicine. 2010;25(9):914-9.

63. Thiebaud P, Patel BV, Nichol MB. Impact of Rofecoxib Withdrawal on Cyclooxygenase-2 Utilization among Patients with and without Cardiovascular Risk. Value in Health. 2006;9(6):361-8.

64. Wang MTM, Gamble G, Grey A. Responses of Specialist Societies to Evidence for Reversal of Practice. JAMA Internal Medicine. 2015;175(5):845-.

65. Zikmund-Fisher BJ, Kullgren JT, Fagerlin A, Klamerus ML, Bernstein SJ, Kerr EA. Perceived Barriers to Implementing Individual Choosing Wisely® Recommendations in Two National Surveys of Primary Care Providers. Journal of General Internal Medicine. 2017;32(2):210-7.

66. Azermai M, Vander Stichele RRH, Van Bortel LM, Elseviers MM. Barriers to antipsychotic discontinuation in nursing homes: an exploratory study. Aging & Mental Health. 2014;18(3):346-53.

67. Hollingworth W, Rooshenas L, Busby J, Hine CE, Badrinath P, Whiting PF, et al. Using clinical practice variations as a method for commissioners and clinicians to identify and prioritise opportunities for disinvestment in health care: a cross-sectional study, systematic reviews and qualitative study. Health Services and Delivery Research. 2015;3(13):1-172.

68. Ibargoyen-Roteta N, Gutierrez-Ibarluzea I, Asua J, Benguria-Arrate G, Galnares-Cordero L. Scanning the horizon of obsolete technologies: Possible sources for their identification. International Journal of Technology Assessment in Health Care. 2009;25(3):249-54.

69. Leggett L, Noseworthy TW, Zarrabi M, Lorenzetti D, Sutherland LR, Clement FM. Health technology reassessment of non-drug technologies: current practices. Int J Technol Assess Health Care. 2012;28(3):220-7.

70. Lesuis N, den Broeder AA, van Vollenhoven RF, Vriezekolk JE, Hulscher M. Choosing wisely in daily practice: a mixed methods study on determinants of antinuclear antibody testing by rheumatologists. Scandinavian Journal of Rheumatology. 2017;46(3):241-6.

71. Massatti RR, Sweeney HA, Panzano PC, Roth D. The De-adoption of Innovative Mental Health Practices (IMHP): Why Organizations Choose not to Sustain an IMHP. Administration and Policy in Mental Health and Mental Health Services Research. 2008;35(1-2):50-65.

72. Robert G, Harlock J, Williams I. Disentangling rhetoric and reality: an international Delphi study of factors and processes that facilitate the successful implementation of decisions to decommission healthcare services. Implementation Science. 2014;9(1):123-.

73. Silverstein W, Lass E, Born K, Morinville A, Levinson W, Tannenbaum C. A survey of primary care patients’ readiness to engage in the de-adoption practices recommended by Choosing Wisely Canada. BMC Research Notes. 2016;9(1):301-.

74. Tatsioni A, Bonitsis NG, Ioannidis JPA. Persistence of Contradicted Claims in the Literature. JAMA. 2007;298(21):2517-.

75. Voorn VMA, Marang-van de Mheen PJ, So-Osman C, Vliet Vlieland TPM, Koopman-van Gemert AWMM, Nelissen RGHH, et al. Designing a strategy to implement cost-effective blood transfusion management in elective hip and knee arthroplasties: A study protocol. Implementation Science. 2012;7(1):58-.

76. Voorn VMA, Marang-van de Mheen PJ, So-Osman C, Kaptein AA, van der Hout A, van den Akker-van Marle ME, et al. De-implementation of expensive blood saving measures in hip and knee arthroplasties: study protocol for the LISBOA-II cluster randomized trial. Implementation Science. 2014;9(1):48-.
