# Supplementary material for: Determinants of the de-implementation of low-value care: a multi-method study
Source: BMC Health Serv Res. 2022 Apr 6;22:450. doi: 10.1186/s12913-022-07827-4 (PMC8985316; doi:10.1186/s12913-022-07827-4)
Supplement: Supplementary file 6 — Additional file 6. Summary of QATSDD scores for included original research articles. [file 12913_2022_7827_MOESM6_ESM.docx]

**Additional File 6. Summary of QATSDD scores for included original research articles**

| **Study Design** | **No. of studies** | **Median Overall Quality Score (IQR)** |
| --- | --- | --- |
| Mixed-methods | 9 | 46% (40%-58%) |
| Qualitative | 13 | 45% (38%-57%) |
| Quantitative | 52 | 52% (45%-57%) |
