# Supplementary material for: Determinants of the de-implementation of low-value care: a multi-method study
Source: BMC Health Serv Res. 2022 Apr 6;22:450. doi: 10.1186/s12913-022-07827-4 (PMC8985316; doi:10.1186/s12913-022-07827-4)

**Additional File 7. Ten most commonly cited determinants of the de-implementation of low-value practices.^a^ A: barriers, B: facilitators.** ^a^Increased size and depth of colour indicate a higher number of citations underpinning the reported barrier or facilitator

**A.**


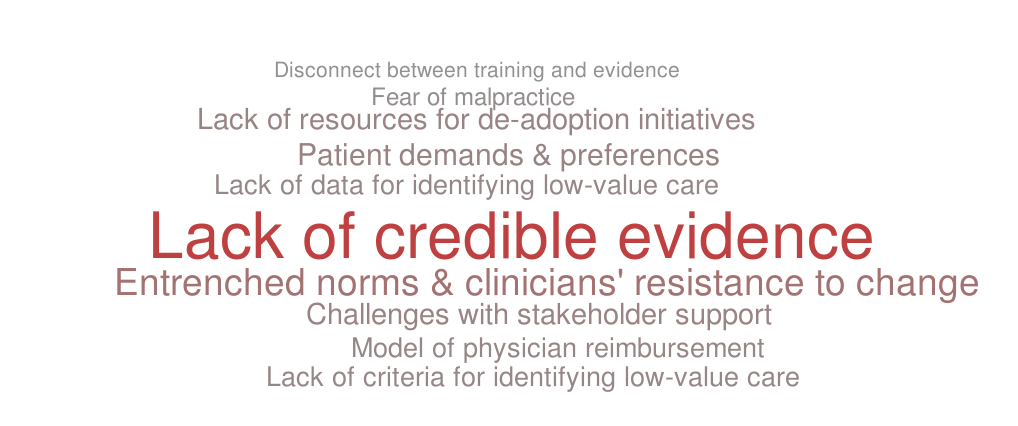


**B.**


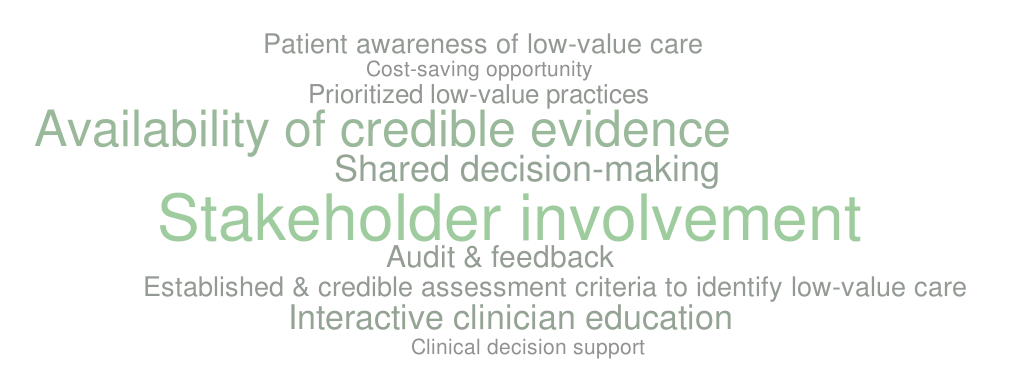

Supplement: Supplementary file 7 — Additional file 7. Ten most commonly cited determinants of the de-implementation of low-value practices.a A: barriers, B: facilitators. aIncreased size and depth of colour indicate a higher number of citations underpinning the reported barrier or facilitator. [file 12913_2022_7827_MOESM7_ESM.docx]
