# Supplementary material for: Determinants of the de-implementation of low-value care: a multi-method study
Source: BMC Health Serv Res. 2022 Apr 6;22:450. doi: 10.1186/s12913-022-07827-4 (PMC8985316; doi:10.1186/s12913-022-07827-4)
Supplement: Supplementary file 8 — Additional file 8. Determinants of the de-implementation of low-value practices mapped to relevant stakeholders. [file 12913_2022_7827_MOESM8_ESM.docx]

**Additional File 8. Determinants of the de-implementation of low-value practices mapped to relevant stakeholders**

| **Barrier** | **Relevant stakeholders** |
| --- | --- |
| Lack of credible evidence to support de-adoption of a low-value practice | Researchers, Clinicians, Decision-makers |
| Entrenched norms and clinicians' resistance to change | Clinicians |
| Patient demands and preferences | Patients |
| Challenges with securing, mobilizing, and maintaining appropriate stakeholder support | Researchers, Decision-makers, Clinicians, Patients |
| Lack of resources (eg, financial, expertise) for de-adoption initiatives | Researchers, Decision-makers |
| Model of physician reimbursement | Decision-makers, Clinicians |
| Lack of commensurate evaluation methods and data for identifying candidate low-value practices | Researchers, Decision-makers |
| Lack of criteria for identifying low-value practices | Researchers, Decision-makers |
| Clinician's fear of malpractice | Clinicians |
| Perceived disconnect between clinical training and evidence | Clinicians |
| Lack of understanding of de-adoption process | Clinicians, Researchers, Decision-makers |
| Perception of risk to patients associated with de-adoption | Clinicians, Patients |
| Lack of political and industry support | Decision-makers |
| Applicability of de-adoption intervention to individual patient/condition | Researchers, Patients |
| Framing of rationale for de-adoption (eg, cost-cutting) | Decision-makers |
| Poor dissemination of evidence and suggestions to de-adopt a low-value practice | Researchers, Decision-makers, Clinicians |
| Clinicians' inability to understand scientific evidence (eg, statistics) | Clinicians |
| Communication gaps between clinicians (ie, continuity of care) | Clinicians |
| Healthcare system that is complex and unconducive to change | Decision-makers, Clinicians |
| Patients unaware of cost of medical tests and treatments | Patients |
| Clinicians' challenges with effectively communicating with patients about low-value practices | Clinicians, Patients |
| Perceived loss of clinical autonomy | Clinicians |
| Lack of alternative tests or treatments if practice is de-adopted | Clinicians |
| Lack of clinical decision support | Clinicians |
| Lack of understanding of barriers and facilitators to de-adoption | Researchers, Decision-makers |
| Small-scale interventions instead of system-level changes | Decision-makers, Researchers |
| Time constraints during patient visits | Decision-makers, Clinicians, Patients |
| Concern with response from insurance companies | Decision-makers |
| Unclear goal for de-adoption intervention | Researchers, Decision-makers |
| **Facilitator** | **Relevant stakeholders** |
| Stakeholder collaboration and communication in development and implementation of the de-adoption intervention | Decision-makers, Researchers, Patients, Clinicians |
| Availability of credible evidence to support de-adoption of a low-value practice | Researchers, Decision-makers, Clinicians |
| Physician-patient communication and shared decision-making about use of targeted low-value practice | Clinicians, Patients |
| Interactive clinician education about targeted-low value practice and/or de-adoption | Clinicians |
| Audit and feedback for clinicians | Clinicians |
| Patient awareness of the targeted low-value practice and need for de-adoption | Patients |
| Established and credible assessment criteria to identify low-value practices | Researchers, Decision-makers |
| Prioritized low-value practices | Researchers, Decision-makers |
| Cost-saving opportunity | Decision-makers |
| Clinical decision support | Clinicians |
| Positive influence from political or industry stakeholders | Decision-makers |
| Performance incentives for clinical staff | Clinicians |
| Clinical champions | Clinicians |
| Evaluation of de-adoption intervention implementation and outcomes | Researchers |
| Medical culture and norms that supports evidence-informed care | Clinicians, Decision-makers, Patients |
| Framing de-adoption as a reallocation of resources (ie, not cost-cutting) | Decision-makers |
| De-adoption process models | Researchers, Decision-makers |
| Implementation of de-adoption intervention at the system-level | Decision-makers, Researchers |
| Infrastructure for accurately measuring use of the low-value practice | Decision-makers, Researchers |
| Assurance that de-adoption will not be harmful to patients | Clinicians, Patients |
| Available alternatives to the low-value practice | Clinicians |
| Multi-modal de-adoption interventions | Researchers |
| Respect for clinical autonomy | Clinicians |
| Value-based insurance design | Decision-makers |
