# Supplementary material for: Determinants of the de-implementation of low-value care: a multi-method study
Source: BMC Health Serv Res. 2022 Apr 6;22:450. doi: 10.1186/s12913-022-07827-4 (PMC8985316; doi:10.1186/s12913-022-07827-4)
Supplement: Supplementary file 9 — Additional file 9. Determinants of the de-implementation of low-value practices mapped to the Theoretical Domains Framework (TDF). [file 12913_2022_7827_MOESM9_ESM.docx]

**Additional File 9. Determinants of the de-implementation of low-value practices mapped to the Theoretical Domains Framework (TDF)**

|  |  | **No. of times cited** | |  | **No. of times cited** | |
| --- | --- | --- | --- | --- | --- | --- |
| **Domain (definition)** | **Barrier to De-implementation** | **Original research** | **Non-original research** | **Facilitator to De-implementation** | **Original research** | **Non-original research** |
| Knowledge (An awareness of the existence of something) | Lack of credible evidence to support de-implementation of a low-value practice | 38 | 52 | Availability of credible evidence to support de-implementation of a low-value practice | 18 | 16 |
|  | Lack of commensurate evaluation methods and data for identifying candidate low-value practices | 4 | 19 | Interactive clinician education about targeted low-value practice and/or de-implementation | 16 | 8 |
|  | Lack of criteria for identifying low-value practices | 9 | 14 | Prioritized low-value practices | 8 | 5 |
|  | Poor dissemination of evidence and suggestions to de-implement a low-value practice | 1 | 7 | Established and credible assessment criteria to identify low-value practices | 10 | 7 |
|  | Lack of understanding of barriers and facilitators to de-implementation | 2 | 1 | Infrastructure for accurately measuring use of the low-value practice | 0 | 2 |
| Skills (An ability or proficiency acquired through practice) | Clinicians' inability to understand scientific evidence (e.g., statistics) | 5 | 3 | Physician-patient communication and shared decision-making about use of targeted low-value practice | 9 | 16 |
|  | Clinicians' challenges with effectively communicating with patients about low-value practices | 2 | 3 |  |  |  |
| Social/professional role and identity (A coherent set of behaviours and displayed personal qualities of an individual in a social or work setting) | - |  | - | - |  | - |
| Beliefs about capabilities (Acceptance of the truth, reality or validity about an ability, talent or facility that a person can put to constructive use) | Perceived loss of clinical autonomy | 0 | 4 | Respect for clinical autonomy | 0 | 1 |
| Optimism (The confidence that things will happen for the best or that desired goals will be attained) | - |  | - | - |  | - |
| Beliefs about Consequences (Acceptance of the truth, reality, or validity about outcomes of a behaviour in a given situation) | Clinician’s fear of malpractice | 10 | 8 | Assurance that de-implementation will not be harmful to patients | 0 | 1 |
|  | Perception of risk to patients associated with de-implementation | 4 | 9 |  |  |  |
| Reinforcement (Increasing the probability of a response by arranging a dependent relationship, or contingency, between the response and a given stimulus) | Model of physician reimbursement | 9 | 15 | Performance incentives for clinical staff | 4 | 5 |
| Intentions (A conscious decision to perform a behaviour or a resolve to act in a certain way) | - |  | - | - |  | - |
| Goals (Mental representations of outcomes or end states that an individual wants to achieve) | Framing of rationale for de-implementation (eg, cost-cutting) | 3 | 6 | Evaluation of de-implementation intervention implementation and outcomes | 2 | 5 |
|  | Unclear goal for de-implementation intervention | 0 | 1 | Framing de-implementation as a reallocation of resources (ie, not cost-cutting) | 1 | 5 |
| Memory, attention and decision processes (The ability to retain information, focus selectively on aspects of the environment and choose between two or more alternatives) | Perceived disconnect between clinical training and evidence | 8 | 6 | Clinical decision support | 4 | 9 |
|  | Applicability of de-implementation intervention to individual patient/condition | 4 | 6 |  |  |  |
|  | Lack of clinical decision support | 1 | 2 |  |  |  |
| Environmental context and resources (Any circumstance of a person’s situation or environment that discourages or encourages the development of skills and abilities, independence, social competence and adaptive behaviour) | Challenges with securing, mobilizing, and maintaining appropriate stakeholder support | 16 | 11 | Stakeholder collaboration and communication in development and implementation of the de-implementation intervention | 21 | 22 |
|  | Lack of resources (eg, financial, expertise) for de-implementation initiatives | 14 | 13 | Cost-saving opportunity | 6 | 7 |
|  | Lack of understanding of de-implementation process | 9 | 5 | Positive influence from political or industry stakeholders | 8 | 5 |
|  | Lack of political and industry support | 6 | 5 | De-implementation process models | 1 | 3 |
|  | Healthcare system that is complex and unconducive to change | 0 | 3 | Implementation of de-implementation intervention at the system-level | 0 | 3 |
|  | Lack of alternative tests or treatments if practice is de-implemented | 2 | 2 | Available alternatives to the low-value practice | 0 | 1 |
|  | Small-scale interventions instead of system-level changes | 0 | 3 | Multi-modal de-implementation interventions | 0 | 1 |
|  | Time constraints during patient visits | 0 | 3 | Value-based insurance design | 0 | 1 |
|  | Concern with response from insurance companies | 0 | 2 |  |  |  |
| Social influences (Those interpersonal processes that can cause individuals to change their thoughts, feelings, or behaviours) | Entrenched norms and clinician resistance to change | 16 | 27 | Patient awareness of the targeted low-value practice and need for de-implementation | 4 | 13 |
|  | Patient demands and preferences | 19 | 9 | Medical culture and norms that support evidence-informed care | 3 | 5 |
|  | Communication gaps between clinicians (ie, continuity of care) | 3 | 5 | Clinical champions | 3 | 4 |
|  | Patients unaware of cost of medical tests and treatments | 2 | 3 |  |  |  |
| Emotion (A complex reaction pattern, involving experiential, behavioural, and physiological elements, by which the individual attempts to deal with a personally significant matter or event) | - |  | - | - |  | - |
| Behavioural regulation (Anything aimed at managing or changing objectively observed or measured actions) | - |  | - | Audit and feedback for clinicians | 4 | 15 |
