# Supplementary material for: Determinants of the de-implementation of low-value care: a multi-method study
Source: BMC Health Serv Res. 2022 Apr 6;22:450. doi: 10.1186/s12913-022-07827-4 (PMC8985316; doi:10.1186/s12913-022-07827-4)
Supplement: Supplementary file 10 — Additional file 10. Determinants identified in stakeholder interviews with exemplar quotations. [file 12913_2022_7827_MOESM10_ESM.docx]

**Additional File 10. Determinants identified in stakeholder interviews with exemplar quotations**

| **Determinants** | **Exemplar Quotations** |
| --- | --- |
| **Barriers** | |
| Applicability of de-adoption to individual patient | “So yeah, it's balancing the evidence that applies to a thousand patients to one patient in front of you who is your problem.” Par 4  “And I think too, you try and look at the evidence and be rational, but in truth here I think the evidence has a lot of limitations in the sense that some of the populations won't apply to the person sitting in front of you.” Par 5 |
| Challenge with securing, mobilizing and maintaining appropriate stakeholder support | “Interpersonal things, I think like if there's other people who have tried this out, the de-adoption and it hasn't worked for them and they start talking to other people, then other people will probably be like, "Oh well this person who I trust and respect tried to try this and it hasn't worked for them or they're really skeptical about it." And then that might influence other people's willingness.” Par 12  “So the other thing around like the actual work, so I'd say when you're facilitating, um, there's often times that, that sort of hierarchical balance that you see on the floor, you oftentimes see in the rooms as well. So there's people that don't really share and want to be honest about how things are done, um, when there's a physician in the room or they feel that what they have to say isn't as important. And so really building up, you know, the unit clerk’s perspective on the fact that they're the hub of all the communications and we don't know what we don't know unless they tell us. Right? And that can maybe make or break a process is really, really important.” Par 2 |
| Clinician's fear of malpractice | “So, I think that could be a way for people to do things just because they're trying to cover all their bases and they make unnecessary referrals or test requests. Just to check these boxes in case they were to get sued.” Par 10  “Because nobody wants to be seen as limiting therapy in a situation where people's lives are immediately at risk. And I think if anybody did question it, unless it came from within the group, it could be turned around and used against them.” Par 11 |
| Clinicians' challenges with effectively communicating with patients | “Sometimes being at the bedside, you could have a family member that's pushing for certain things, whether it's because they read it on the internet or they're a physician themselves or a nurse or have some type of background. But sometimes they can be a bit pushy and I guess it just depends on the physician or the nurse to educate them like, Hey, this is good or this is bad and this is why we're doing it. But that doesn't always happen, just because it's so busy there and a lot of times they don't have time.” Par 10 |
| Clinicians' inability to understand scientific evidence | “The other is their willingness or ability to access the information that they need to change. So again, some people are computer illiterate even though they're working in healthcare. So if you think a symptom is not presented in the way that they can or would like to access it, they're less likely to change. Yeah, I'd say being mindful of people's learning needs. Some people, if they have to do it on their own and they have busy lives for one reason or another outside of the work environment may not ever learn what they need to know to change.” Par 11  “One of the barriers is just the sheer amount of evidence that's coming out in any given topic. So trying to consume that, digest it and then synthesize things out. I have a couple of areas of interest of my own. I find it challenging to keep up in my own little niche areas. So to have that kind of depth of knowledge in every single area to the point where you can make a recommendation on whether as a system we should be de adopting things, it's really challenging.” Par 15 |
| Communication gaps between clinicians | “I think... barriers in coordinating between different stakeholders... We don't have- I mean we have grand rounds and things where we talk about these things, but we don't have for, uh- like an interdisciplinary forum, for like, really discussing these issues. Um, that I've really seen anyway.” Par 3  “And even when I think about the [states institution where they are is located], it's hard for me to meet with everyone who works at the [states same institution where they are is located], because I think people are stretched out over the city. So, if I have a business meeting, I only ever see half the people. So that coordinating and disseminating them for at least the [states institution where they are is located] is quite difficult to do.” Par 5 |
| Entrenched norms and clinicians' resistance to change | “I kind of feel like it's generational, if that particular thing is something that they grew up doing in their practice and they'd been doing it for 20 years and now they're saying that it's not beneficial anymore. I think it's hard to get people to change once they've been doing it for so long, 10 years, 20 years, whatever. So, there's like a level of comfort with the old way versus the new way.” Par 10  “I think the biggest reason why people use any practices that are either low-value or even potentially harmful is because it's been so ingrained in the culture. There's many examples of that, going from daily blood work in the ICU to using heparin routinely for CRRT, even though we have good evidence to show that citrate a better anticoagulant. But it's the culture of certain units to use certain practices, and a familiarity with those practices that I think is the biggest barrier to change.” Par 17 |
| Framing of rationale for de-adoption (e.g., cost cutting) | “The one thing about making change is often change is framed as a cost saving measure, and sometimes that is useful and people are behind it and you get it. But then you have to be careful with that because it's something that's framed as just as a way to save money. I think there is a perception that care will suffer if it's just a financially driven change, or at least the perception of a financially driven change. So even if something has no value and is expensive and there's really no reason to do it, it's hard to frame it as well, we can save money by adopting this change.“ Par 13  “Um, so, um, people have a hard time thinking about what they've done for years as being harmful, because there's like a personal element to that, right? You mean I've been doing harm to people for the last like 5, 10 years? Or 30? Um, so there's that personal element, uh, that sort of identifies with- with what you've always done, that's like part of who you are as a physician. Um, and then to be told that that's potentially ineffective or harmful, you know, there's prob- there is like an ego and personal element to that” Par 3 |
| Healthcare system that is complex and unconducive to change | “Oh, so number one is the culture that exists within healthcare and medicine specifically. So people tend to be very conservative in change.” Par 11  “Um, like if you have, like, a specific physician who absolutely refuses to give up his low value practice. Well, then, for that ICU week your efforts dead in the water. It's like, um, huge power differentials in critical care, and, um, and it comes from the top, so, um. And I think, um, that's something we haven't talked about is the fact that in critical care it's like a week at a time, um, that the staff change, um, and so even if you do successfully de-adopt with a few of the staff you can have a few that refuse.” Par 3 |
| Lack of alternative tests or treatments | “If one particular therapy is ineffective or harmful, but somebody feels like they have to do something, then what do they replace it with, you know?” Par 3  “I think too, in maybe critical care, specifically when you have people who are dying in front of you, you kind of have that reflex feeling to do whatever you can to try and help, even though you know necessarily it doesn't change anything, but you have nothing else to do.” Par 5 |
| Lack of commensurate evaluation methods and data for identifying low-value practices | “And there's been lots of things that have taken numerous studies before we've actually figured out the best way to use them. And so if you de adopt too early, the fear of de adopting too early at the risk of throwing something away that might be beneficial, I think that's a big barrier.” Par 15  “And so it's like, "Oh well we should do that because of this." And it's like, "But do we have any information to help us understand if it's actually in fact that if we think it's the most expensive procedure that we're using, is it in fact low valid care?" No.” Par 2 |
| Lack of credible evidence | “So I think the challenges with de-adopting things in critical care are number one trials or studies that show things are ineffective may or may not be inclusive of critical care, critically ill patients. If there are studies that look at critical ill patients specifically, again, sometimes it's easy to overstate the population that's being tested in a lot of these studies and therefore completely de adopt things when in fact the evidence may not have tested certain populations.” Par 15 |
| Lack of political and industry support | “Well, we'll have to go through committee. Oh, we'll have to do this. We'll have to do that. And it's also mostly answered by a no. Oh, no, we don't do this.” Par 8  “This needs to be driven by physicians, not driven by government policies. Government won't do it, right? They'll tell us that they want us to spend less but they won't actually drive it.” Par 16 |
| Lack of resources (e.g., financial, expertise) | “And then if they don't have the resources to be able to make the change as well, it would be another barrier. Either time resources or personnel resources or equipment that they required to do that.” Par 12  “What are the results? I want to know per patient how many less of that are we doing. I don't have any of that. And it would be so simple, but we're not equipped.” Par 8 |
| Lack of understanding of de-adoption process | “So some of the barriers would be if staff didn't understand what was going on and why they were doing it. A rollout that happens too quickly. Not enough time, not enough explanation, not enough support. Things like that.” Par 14  “Even to study these things, the activation energy is so high, um, because people are like "why are you studying something we already do?". So, getting, like, getting funding to study them, um, and then actually studying them, and then fighting to convince people that your results are valid and, um, then driving de-adoption efforts.” Par 3 |
| Model of physician reimbursement | “I would say in very rare instances, billing, but I would say that's a very rare instance. I don't think, at least from my reading, there's very little data to support that, that billings drive inappropriate care in this context.” Par 16  “I don't get paid to go to meetings. So that to me has been like, and I totally get it, um, on some level, but on another level, I don't know if I do because I think the work that we're talking about in deciding on directly impacts your job. And so, and it's such a critical job and it's such an important, so whether or not whether we create, I don't know... Yeah. And then it just comes into the compensation, conversation.” Par 2 |
| Patient demands and preferences | “Sometimes being at the bedside, you could have a family member that's pushing for certain things, whether it's because they read it on the internet or they're a physician themselves or a nurse or have some type of background. But sometimes they can be a bit pushy and I guess it just depends on the physician or the nurse to educate them like, Hey, this is good or this is bad and this is why we're doing it. But that doesn't always happen, just because it's so busy there and a lot of times they don't have time.” Par 10  “Oh, so, patients wanting to think that they can... For patient or family reasons, right. So, they want to continue to do, to quote on quote do everything what we might not be able to get them to that attainable goal of getting them better.” Par 16 |
| Patients unaware of cost of medical tests and treatments | “So, the way to reframe that would be to say if you'd pay for this test, and we've often had these discussions that where people order tests and we've often said to somebody, "Okay, well if you were to pay for that yourself, would you order that test on yourself?" And they say, "Well no, that would be stupid." And then, "Well, why are you ordering it on them?" Right. So, patients and families actually had to physically pay for the test like they do in the States, then they would be more cognizant to see if there's any value in that test. But in Canada where everything is perceived as free, they're not as focused on value. So, they're actually a little bit, in my mind, they're actually not the people we need to be talking about because where's it's free people will say, "Well, it's free. Give me whatever I want." And that's part of our problem.” Par 16 |
| Perceived disconnect between clinical training and evidence | “"That's not part of my role. I've never been taught how to de-adopt,"” Par 1  “I think really that it goes back to this idea of, this is how we were trained, this is what we know, these are the tools that we have. So, I think it's really hard once you learn something the first time to change.” Par 13 |
| Perceived loss of clinical autonomy | “Physicians put a high value on autonomy, so if you try and tell them to do things differently or they can't do things, they often will oppose that direction because it is perceived as infringing on their autonomy.” Par 11  “I think people have very good reasons for making the specific choices they make with individual patients and, um, and externally, um, mandating that people do one thing or another is not, um, I think people wouldn't take kindly to that.” Par 3 |
| Perception of risk to patients | “Because nobody wants to be seen as limiting therapy in a situation where people's lives are immediately at risk.” Par 11  “So, I think the, um, the issue really is that the stakes are so high, and a lot of other, um health care settings, you know, if you do or don't do something it might have minimal effect on the patient, and, um, like it might change like a secondary outcome but it's not gonna change whether they live or die. Um, where as in critical care because the patients are so sick, I think there's this real temptation to fall back on what we know and what we do, rather than making the leap to change practice, um, unless there's like, um, you know, one A level evidence, um, uh, to really suggest that that's the case.” Par 3 |
| Poor dissemination of evidence and suggestions | “I think we often don't present enough data or all the data we could in a way that is meaningful to physicians and other healthcare providers.” Par 11  “So if data is told to a certain group that, "This is what we found." And that group doesn't believe in it, then it's going to be very difficult to prove to them that that should be the way.” Par 14 |
| Small-scale interventions instead of system-level | “Well we just thought it would be a good idea to just put up a banner, right?" You can just see where this is going. And it's like, "Okay, well we know those types of things are oftentimes like we do on our computers." Click, click, click and people ignore them.” Par 2  “... but there is also, but in terms of actual, "What are we going to use?" It's usually, I think it generally, it just falls through the sieve and it ends up being a poster or a picture or a post or an email or a, yeah.” Par 2 |
| **Facilitators** | |
| Assurance that de-adoption will not harm patients | “Other things that are important are having people who have the background and capacity to do the work that needs to be done to support de-adoption in our changing practice; whether it's de-adoption or adoption, people won't change unless they're convinced that it's in the best interest of patients to change or the best interests of how we're delivering services, even if it's not at an individual patient level. So that that takes a bit of expertise and work. Work takes time.” Par 11  “And the reason the red tape is important is because it's sort of the safety of the patients and it's so that you can actually get a 360 degree view of the implications of removing something.” Par 15 |
| Audit and feedback for clinicians | “Actually showing people what they do, because I think, um people have a sense of how they practice, um on an individual patient level, but they don't see where they fit into the broader scheme of physicians. Like where they lie on the bell curve of antibiotic prescription or Haloperidol, uh, use in-in delirium or whatever else. But if you take data and show them where they sit and show them if they're an outlier or, um, that may, um, help people to shift their practice more.” Par 3  “Items that the department feels strongly about de-adopting, they should consider putting that in the performance indicators, and the individual physicians should get feedback on how well they're doing on that. And so if I hear that I'm the only one not de-adopting a practice. I think there's a really strong impedance to remove that from my practice.” Par 7 |
| Availability of credible evidence | “Basically giving them a rationale for changing their practice. I think if people are able to be provided with specific data relevant to their own practice they're more likely to change as well.” Par 12  “I would say, one you need to have the research behind the de-adoption of something, something that clearly shows it's not working. And something a little bit more tangential than anecdotal evidence, and I say that because there is always pushback to change, and I think that it's hard for people to argue with good research that shows that something doesn't have much of a value.” Par 13  “So anytime anything is coming out as a big guideline recommendation, if it comes out from a high-impact journal, those are the... If it says that something is harmful or something is beneficial, or something has recommended grade-A evidence. Those are big drivers.” Par 7 |
| Available alternatives | “So what we did is we determined that the value of the ECG post open heart surgery was really immediate post op. That was the big bang for the buck. So we said, "Yeah, we'll invest in this because it matters if there's ischemia post open heart surgery, we want to have the best tests." But the next day, the value added is not that great, so we stopped asking the lab to come and do the ECG and we train our nurses to do the ECG using an ECG modalities that could be done through the monitor that the patient is using, the ECG monitor.” Par 8  “So we used to do blood gasses, lactate level, routinely we used to do them the next day in every cases. So we streamlined things and what we did is for a lot of those blood tests, instead of doing a blood gas, which is about $30 a test, we will order electrolytes and a CBC. And those are pennies. The difference is that the blood gas, you'll have the result right away and the electrolytes and the CBC takes about 30 minutes to an hour.” Par 8 |
| Clinical champions | “Facilitators. I think... people who have skin in the game. Like, people who, um, who believe in the de-adoption effort. So whether that be residents, fellow staff, um, um, champions of the de-adoption process, um, uh, pushing it forward” Par 3  “I think facilitator, probably having champions of kind of that people who really promote and be the kind of go to people for whatever type of intervention that you're proposing. I think that's really important, and consistency and presence.” Par 5 |
| Clinical decision support | “If it's a change in people's clinical behavior, usually you need some sort of either decision support or controls on ordering, ideally backed up by some policy and procedure, or that's actually, it can be a form of decision support, usually some sort of decision support and or other controls.” Par 11  “And then checking in with people as well. Like, "how's that going for you? What things are, what challenges are you encountering, what's been your experience?" And then kind of help them with some problem-solving along the way. Those things can be effective as well.” Par 12 |
| Cost-saving opportunity | “Well, probably one of the biggest things with critical care is it's such a resource-heavy specialty, due to the nature of the critically ill patients, that de-adoption of one small test or therapy could result in significant cost savings. So I think there's the opportunity to scale whatever may be de-adopted, as far as not being valuable, maybe harmful, scale that to having a larger effect than perhaps some other specialties.” Par 17  “Yeah, it basically posted on things that we use quite frequently or maybe using too much of or something you would realize costs a certain amount, and they post the price on it. Just, so it triggers your mind to be like "Do I really need this, is this important or am I just using it ‘cause I like it?"” Par 9 |
| De-adoption process models | “I think the ones that I've seen that have been relatively successful have been a combination of approaches, but policy, order sets, education, and then there's been some sort of reminders and audit and feedback.” Par 15  “I think there should be two processes invented or characterized. One for the simple, rapid projects locally owned, locally managed. And then you have a more formal process for the bigger projects where you need communal marriage of a lot of departments where the numbers are big, where the impacts will matter. “ Par 8 |
| Established and credible assessment criteria | “So whether that be like retrospective data or prospective, um, just having the... robust system of data to prove the point that it's actually ineffective, and then replicating those results, and in other places to prove that it's not the, um, the, um, idiosyncrasy of the data, or, um, a local phenomenon, that it's a, um, broader, um, phenomenon that actually will impact practice, um, more broadly. Um, and then after that the dissemination, uh, of those results, um, and the chance to hash things out at conferences and meetings and things, um, before people decide whether they're actually gonna believe it.” Par 3  “Identification and then so also that the method you're going to use to study the change and study the impact of that change, you need an expert that will reassure that your efforts are not worthless. Because it's easy to say, "Oh, okay, we'll do this," and then at the end they say, "Oh, my God. We forgot to sample this. I'm not going to review a thousand files." Oh, well, okay. So you need an expert that would guide you with expertise if there is a potential adverse event positive or negative. But if it's streamlining, we don't need help. We need common sense.” Par 8 |
| Evaluation of de-adoption intervention implementation and outcomes | “Data... Has got to be data, not necessarily studied data, but it's got to be good data that's continually checked and monitored to see if you're actually what you're doing. And then when you make the change, is there an effect, the positive or the negative on outcomes and is it cheaper and it's got to be continuous data and it's got to be consistent across multiple organizations so you could compare and contrast.” Par 16  “Again, it’s not about just implementing a solution and moving away it's implementing it and continuing to measure it to understand if you actually did change for the better and then also ensuring you didn't affect something else negatively. And then going back to understand by that very measurement, whether you're reflecting it positively or negatively and or sustaining your change, you can sort of understand if you need to intervene again in a different way or if... Right it's kind of that whole thing.” Par 2 |
| Framing de-adoption as a reallocation of resources | “So an example would be if a lab test is five dollars and somebody's critically ill in an ICU, it doesn't sound very compelling that you shouldn't order that test for five dollars. But if we order 10 unnecessary five-dollar tests on every patient, that's $50 a day, times 20 patients in the unit is $1,000 a day, times three hundred sixty five is $365,000. and if you can contextualize that, Jeez, we couldn't buy that new piece of equipment, the ultrasound machine you wanted to use at the bedside because it's $70,000 and we don't have that money. But if we saved money, we could spend money sort of thing. So I think sometimes it's got to be framed properly, where it's not just about the money, or it's just not about the one test that you may want to order once a day on a patient, but part of something that's bigger, than people better appreciate it's importance.” Par 11  “We cut this stuff that we didn't, that was not helping very much and we're able to reallocate it to something that we think helps a lot and we see it. But if you, in at the institutional level, if you take away this pile and it vanishes, then little reward. Like a portion of those funds I think should go back to something that's meaningful to the unit. A few cost savings or something that you can actually see. Just kind of make it a nice positive. Like you guys have done this, you've done better care, and it results in this much less problem, and then not just that you saved this much and now you've been able to re allot these funds to this, which you needed and then or maybe improve.” Par 4 |
| Implementation of de-adoption intervention at system-level | “And then other facilitators, again, if there's reasonable reminders in place for people that aren't super annoying and can cause resistance as well. And again, reasonable policies and procedures in place that help support rather than obstruct change I think also can be helpful in leadership support for the change, de-adoption as well for people to come together and share experiences of what's been working for them and what's been challenging so they can talk about that and come up with solutions together.” Par 12  “I think they probably ranged from, things like, if you don't want something used in your unit you just remove it, or not stock it, or make it exceedingly difficult to get. I think the other thing, other ways I think that there's certainly education and promotion or advertising within the unit or as a department as a whole.” Par 5 |
| Interactive clinical education | “And facilitators would be like having education in an effective way as opposed to someone just standing up in front of the room and doing a PowerPoint presentation and more interactive types of learning experiences and opportunities to practice having some coaching and feedback are other facilitators. I think in terms of, if people are running up against challenges, talking with them about like how to maybe address some of those challenges in de-adoption.  Par 12  “I think education is a large one as well. I find some things on our unit, we've got like a formal education or someone will come around and you have to even initial that you went through this like little teaching scenario, and I find people take that more seriously than if just an email goes out and says like, "Hey stop doing this or we don't need to do that anymore". I find if it's a little bit more formal education, people follow along a little bit more. I think maybe just how it's presented and the seriousness behind it could be a factor.” Par 9 |
| Medical culture and norms that support | “Another thing that I think with change management that can be helpful is if you ask people to identify goals for themselves, like a commitment to change. That's something that we often do in faculty development workshops is "now that you've had this education, what will you change in your practice two months from now?" And then we're going to call you, follow up with you at that time and to see if you've actually made that change and if they haven't been, what have been obstacles and how can we problem solve with you to make things make that change possible.” Par 12  “Well, I would consider one contextual factor. Look at the tightness of the group of providers. So does this group of providers get together and share opinions in journal club? Do they help coach each other in terms of other practice? So for example, if I'm struggling with procedure, do I reach out to other providers around me to help learn? So in all of the other ways that we practice are we are homogeneous and helpful group. If we're close, then we're more likely to de-adopt all together.” Par 7 |
| Multi-model de-adoption interventions | “I think various ways. I think they probably ranged from, things like, if you don't want something used in your unit you just remove it, or not stock it, or make it exceedingly difficult to get. I think the other thing, other ways I think that there's certainly education and promotion or advertising within the unit or as a department as a whole. And certainly other functions like trying to take advantage of the electronic record, and so prompts and FCM orders. I think that a lot of personnel things like we're trying to say delegate specific members of the team and make it their responsibility to remind or bug the physicians about various aspects of things.” Par 5 |
| Patient awareness | “So I can imagine myself being a patient and going, well, do I really need that lab today? I've already had a normal hemoglobin for three days in a row, can I just let you know if I feel off. And so they can advocate for themselves in that way.” Par 7  “I think safety is stuff that isn't obviously low value and de-adoption would come from safety issues as well. And, uh, why aren't we sharing that? Like if people don't know what the bedside, when they're caring for the, when they're with their families, you know, something could be going on that they're not, if they're aware of it, they could be the, that, you know that indicator that lets everybody know.” Par 2 |
| Physician-patient communication and shared decision-making | “I think there's certainly benefit to having patients and family members involved. I think there's going to be a lot of things that they don't know anything about and there's clear clinical benefit. I think probably where they would be most beneficial, are for traditional clinical outcomes there's Equipoise, and perhaps they can provide a lens on which technique or which test might be a better experience for the patient.” Par 15  “What we've found through basically all of our patient-engaged studies, and patient-engaged work is that what we as healthcare providers perceive as high-value and importance is not necessarily the same as what patients perceive. So I think it goes hand-in-hand there, and that they should be partners in this, and have a seat at the table, and be able to themselves express what they may feel as valuable and important to keep in terms of healthcare provision, as well as what has no value to them and what we can de-adopt.” Par 17 |
| Positive influence from political or industry stakeholders | “And also for the leadership of that unit to be involved as well. So the unit medical director and unit nursing manager as well, and that they're aware and involved and engaged in it, in the process as well and then obviously, the people who are on that committee, would be the people who are for sure brought in.” Par 12  “I think probably in terms you always want the support of your general leadership and admin operations side of it.” Par 5 |
| Respect for clinical autonomy | “I think it's successful because they can still be done and accessed when they are needed.” Par 6  “I don't have to go through tons, layers and layers of people saying, "Well, I think we should have this kit assessed by a large group." And between you and me, I really don't care what the nurses think when it comes to a surgical kit that I'm going to use, okay? This is within my area of expertise. That's how they feel upstairs in CVICU. Smaller group, leadership is trusted. They know that if it doesn't go well and the kit is no good, I'll assume my responsibility. I'll change my mind. I'll change it. I'll listen to other people, but it is my responsibility.” Par 8 |
| Stakeholder collaboration and communication | “A way to facilitate that is to ensure that you have a good, broad stakeholder engagement with individuals from all involved groups. So from the prescriber or physician group, the bedside nursing group, the educator group, and the administrator group. And to have those people, especially in the early phases of de-adoption after a practice has been identified as being low-value, to really put in the person hours at that time to ensure a consistent, repeated, and the same message to all groups.” Par 17  “So this was determined, again, in collaboration with the unit. So we have a committee that meets every two months and we decide on new projects and collectively, the nurse teachers, the nurse in charge, the bedside nurses, the cardiac surgeon, the intensivist, all thought that this was value added and we're saving more than $80,000 a year with this.” Par 8 |
